# Second-Generation Aldosterone Synthase Inhibitors for Hypertension: A Bayesian Meta-Analysis of Randomized Trials

**DOI:** 10.1016/j.jacadv.2026.102621

**Published:** 2026-02-27

**Authors:** Flavia Queiroga, Beatriz Araújo, André Rivera, Leo Consoli, Aditi Ujjawal, Ensieh Sadat Mansouri, Maria Antonia Costa Cruz Akabane, Nelson I. Barrera, Ana Beatriz Valverde Ramos, Asad Iqbal, Marcelo Braga, Anna K. Krawisz, Paula Dibo, Deepak L. Bhatt

**Affiliations:** a Division of General Internal Medicine, Department of Medicine, Emory University, Atlanta, Georgia, USA; b Department of Medicine, Universidade Nove de Julho, São Bernardo do Campo, Brazil; c Department of Medicine, Universidade Federal da Bahia, Bahia, Brazil; d Division of Cardiology, Beth Israel Deaconess Medical Center, Harvard Medical School, Boston, Massachusetts, USA; e Department of Medicine, University of Medical Sciences, Tehran, Iran; f Department of Medicine, Universidade Federal de Juiz de Fora, Juiz de Fora, Brazil; g Division of Cardiology, Columbia University Medical Center, New York, New York, USA; h Department of Medicine, Pontificia Universidade Catolica do Parana, Parana, Brazil; i Department of Medicine, Bacha Khan Medical College, Mardan, Pakistan; j Department of Medicine, Universidade Federal do Rio de Janeiro, Rio de Janeiro, Brazil; k Mount Sinai Fuster Heart Hospital, Icahn School of Medicine at Mount Sinai, New York, New York, USA

**Keywords:** aldosterone, aldosterone synthase inhibitors, chronic kidney disease, hypertension

## Abstract

**Background:**

Second-generation aldosterone-synthase inhibitors (ASIs) may offer a novel treatment for hypertension.

**Objectives:**

The objective of the study was to assess the efficacy and safety of ASIs in this clinical setting.

**Methods:**

We searched major databases for randomized controlled trials (RCTs) assessing ASIs (baxdrostat, lorundrostat, and vicadrostat) in patients with hypertension. For efficacy outcomes, mean differences (MD) with 95% credible intervals (CrIs) were estimated using a Bayesian random-effects model. For adverse events, OR with 95% CrI were estimated using a Bayesian binomial-normal hierarchical model. The protocol was registered in Prospective Register of Systematic Reviews (CRD420251132306).

**Results:**

Eight RCTs were included (n = 3,369; 2,430 [72%] randomized to ASI). ASI reduced systolic blood pressure (SBP) (MD: –6.7 mm Hg; CrI: −8.78, −4.59; τ^2^ 3.24), diastolic blood pressure (MD: -2.09 mm Hg; CrI: −3.68, −0.44; τ^2^ 1.44), and hypertensive urgency (OR: 0.36; CrI: 0.13, 0.90; τ^2^ 0.07) compared with placebo. There was no difference in all-cause mortality (OR: 0.45; CrI: 0.06, 3.20; τ^2^ 0.10) or adrenal insufficiency (OR: 0.5; CrI: 0.1, 3.1; τ^2^ 0.3) between groups. However, ASIs increased the odds of hyperkalemia (OR: 7.1; CrI: 3.56, 15.2; τ^2^ 0.23), hyponatremia (OR: 2.6; CrI: 1.25, 5.98; τ^2^ 0.1), and hypotension (OR: 3.28; CrI: 1.43, 8.16; τ^2^ 0.1). In subgroup analysis, the probability of achieving a clinically meaningful reduction in SBP (MD < −5 mm Hg) was 87.5% with baxdrostat and 94.3% with lorundrostat.

**Conclusions:**

Second-generation ASIs had a high likelihood of a clinically significant reduction in SBP compared with placebo. However, hyperkalemia, hyponatremia, and hypotension were more frequent with ASIs.

Hypertension remains the leading modifiable contributor to cardiovascular morbidity and mortality, with substantial impact on the incidence of stroke, heart failure, and chronic kidney disease (CKD).^1^, ^2^, ^3^ Despite the availability of multiple antihypertensive drug classes, a considerable proportion of patients fail to achieve adequate blood pressure (BP) control.^4^^,^^5^ The renin-angiotensin-aldosterone system (RAAS) plays a pivotal role in BP regulation, and mineralocorticoid receptor antagonists (MRAs) are a cornerstone of RAAS-targeted therapy in this population.^6^^,^^7^

However, the use of MRAs, particularly steroidal agents such as spironolactone and eplerenone, is often limited by adverse effects including hyperkalemia and sex hormone–mediated side effects.^8^ Even nonsteroidal MRAs such as finerenone, although better tolerated, yield only modest BP reductions with potential compensatory aldosterone increases that attenuate therapeutic benefit.^9^

Second-generation aldosterone synthase inhibitors (ASIs) offer a distinct approach by selectively inhibiting the terminal enzyme in aldosterone biosynthesis.^10^ Unlike first-generation ASIs, which also inhibited cortisol production, newer agents such as baxdrostat, lorundrostat, and vicadrostat demonstrated improved selectivity and tolerability in early-phase trials.^11^, ^12^, ^13^ By targeting aldosterone synthesis directly, ASIs may circumvent the limitations of receptor blockade and mitigate aldosterone-mediated fibrosis, inflammation, and oxidative stress.^14^

Although previous meta-analyses have suggested potential benefits of ASIs, their conclusions were based mainly on first-generation agents.^15^^,^^16^ The efficacy and safety profile of second-generation ASIs remains less well established. Several recent double-blind, placebo-controlled, randomized controlled trials (RCTs) evaluating second-generation ASIs have reported promising results.^17^, ^18^, ^19^, ^20^ Therefore, we performed a Bayesian meta-analysis of RCTs to evaluate the efficacy and safety of second-generation ASIs in patients with hypertension. This approach allowed us to incorporate prior beliefs with our data, quantify the probability of clinically meaningful BP reductions, and compare effects at the drug level, providing a clearer interpretation of treatment benefit and differences between agents.

## Materials and methods

This systematic review and study-level meta-analysis followed the Cochrane Collaboration Handbook for Systematic Reviews of Interventions and the Preferred Reporting Items for Systematic Reviews and Meta-Analyses statement.^21^ The protocol was registered at the International Prospective Register of Systematic Reviews (CRD420251132306). Institutional Review Board or ethics committee approval was not required, as this study synthesized data from previously published studies and did not involve analysis of individual-level patient data.

### Data source and search strategy

We searched PubMed, Embase, Cochrane, and ClinicalTrials.gov from inception to August 2025. The search terms included “aldosterone synthase inhibitors”, “baxdrostat”, “lorundrostat”, and “vicadrostat” (Supplemental Table 2). Two authors (B.A. and F.Q.) independently screened titles and abstracts and evaluated the articles for eligibility based on the prespecified criteria. Discrepancies were resolved through consensus among authors.

### Study eligibility

We included studies according to the following criteria: 1) phase 2 or 3 RCTs; 2) enrolled adults with hypertension; 3) compared a second-generation ASI (baxdrostat, lorundrostat, or vicadrostat) vs placebo; 4) double-blind design; and 5) reported at least 1 of the outcomes of interest. For trials that evaluated ASI therapy together with sodium-glucose cotransporter-2 (SGLT-2) inhibitors, only monotherapy groups were considered. If multiple time points were available, we extracted each study’s primary endpoint. No studies were excluded based on language.

### Outcomes

Our efficacy outcomes were changes from baseline in: 1) systolic BP (SBP); 2) diastolic BP (DBP), and 3) achievement of SBP target. Safety outcomes included the incidence of: 1) serious adverse events; 2) drug-related serious adverse events; 3) all-cause mortality; 4) hyperkalemia; 5) severe hyperkalemia (≥6.0 mmol/L); 6) hyponatremia, 7) hypotension; 8) adrenal insufficiency; 9) any arrhythmia; and 10) hypertensive urgency, as defined per each included study. BP assessments were based on the method reported in each trial.

### Statistical analysis

#### Efficacy outcomes

We applied a Bayesian random-effects model, combining prior information with the observed data to obtain the posterior distribution of the pooled effect.^22^ This model was selected to accommodate expected clinical and methodological variability between studies. Unlike a fixed-effect model, which assumes a common underlying effect, the random-effects incorporates between-study heterogeneity, resulting in intervals that reflect both within-study uncertainty and between-study variability.

In the primary analysis, mean differences (MDs) were modeled using a normal likelihood within a normal-normal hierarchical model with pooled effect (mu) and between-study heterogeneity (tau). We applied vague priors for mu ∼ Normal (0, 510) and weakly informative priors for tau (SBP, Half-Normal [1.67]); DBP, Half-Normal [1.6]).^23^

Our model generated posterior distributions for the overall effect and the between-study heterogeneity. We summarized the results as pooled MD and OR with 95% credible intervals (CrIs). For continuous outcomes, we estimated posterior probabilities for each treatment comparison and prespecified clinically relevant thresholds of −5 mm Hg for SBP and −2 mm Hg for DBP.^24^^,^^25^

#### Safety outcomes

Given the low frequency of safety events, we used a binomial-normal hierarchical model suitable for sparse binary data.^26^ In this framework, study-specific log(OR) are assumed to arise from a normal distribution centered on the overall treatment effect (theta), with between-study heterogeneity parameter (tau). We applied a weakly informative prior for theta ∼ Normal (0, 2.82) on the log-OR scale, corresponding to an upper bound of delta = 250 on the OR scale, and a weakly informative Half-Normal (0.5) prior for tau. Results are reported as OR with 95% CrI. Markov chain Monte Carlo sampling was run with 4 chains and 2,000 saved iterations (1,000 burn-in).

All analyses were performed in R (R Environment; version 4.5.0) using the bayesmeta package with default parameters for efficacy outcomes, which applies the DIRECT algorithm to fit random-effect meta-analyses.^22^^,^^27^ Safety analyses with rare events were performed using the MetaStan package.^26^ Further details on models and priors are provided in the Supplemental Methods. We explored the potential for publication bias by visual inspection of funnel plots and Egger test for SBP outcome.

#### Between-study heterogeneity and prediction

Heterogeneity was quantified using the posterior distribution and the between-study standard deviation (τ).^23^^,^^28^ We derived posterior predictive distributions to characterize the range of true treatment effects expected in hypothetical future studies similar to those included, accounting for uncertainty in both the pooled effect and heterogeneity.^28^^,^^29^

#### Subgroup and sensitivity analysis

Subgroup analyses stratified by drug type (baxdrostat vs lorundrostat) and leave-one-out sensitivity analysis were performed for main efficacy outcomes. Sensitivity analysis for overall results and posterior predictive distribution for the between-study heterogeneity parameter was performed with different priors for change from baseline in SBP, including a skeptical prior for the overall effect centered at zero with a narrower standard deviation.

#### Dose-response analysis

A dose-response relationship was evaluated using the Bayesian restricted cubic splines model.^31^ Treatment effect was regressed against the drug's dose using binomial likelihoods and noninformative priors, capturing linear and nonlinear relationships. Three knots were placed at the 10th, 50th, and 90th percentiles of the observed dose distribution. The model was fit using Markov chain Monte Carlo sampling with 3 chains (100,000 iterations; 10,000 burn-in). From the model's posterior we estimated mean effects at prespecified doses, with 95% CrIs, and estimated probabilities of clinically important benefits/harms at each dose.

#### Quality assessment

A risk of bias assessment was performed with the Cochrane Collaboration’s tool for assessing risk of bias in randomized trials (RoB-2)^30^ for RCTs by 3 independent authors (A.B.V.R., M.A.C.C.A., and N.B.). Any disagreements were resolved by consensus.

## Results

### Study selection and characteristics

**Figure 1:**
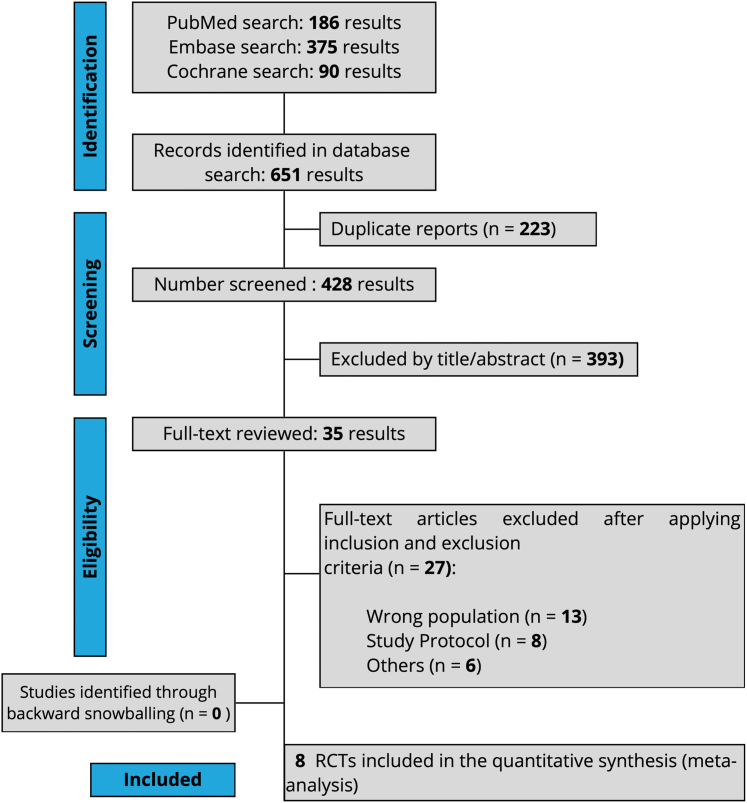
**Preferred Reporting Items for Systematic Reviews and Meta-Analysis Flow Diagram of Study Screening and Selection** RCT = randomized controlled trial.

Our systematic search yielded 651 potential results (Figure 1). After removing duplicates and ineligible studies, 35 remained for full-text review. Eight RCTs^11^, ^12^, ^13^^,^^17^, ^18^, ^19^, ^20^^,^^32^ met the eligibility criteria. All included trials were phase 2/3, multicenter, RCTs conducted across diverse geographic regions.

A total of 3,369 individuals were included, of whom 2,430 (72%) received ASI. Among those, 1,050/2,430 (43.2%) received baxdrostat, 1,165/2,430 (47.9%) received lorundrostat, and 215/2,430 (8.9%) received vicadrostat. The mean age was 62.2 years (SD: 10.6), 1,911/3,332 (57.4%) were men. A total of 828/3,369 (24.6%) participants identified as Black. The baseline SBP was 146.4 mm Hg (range: 135.0, 151.2), and the median follow-up duration across studies was 12 weeks (range: 8-26). Diabetes was present in 1,296/3,120 (41.5%) participants, and the mean estimated glomerular filtration rate across studies was 85.5 mL/min/1.73 m^2^ (range: 44.7-91.0). Across trials with available data, 80 to 100% were on background angiotensin-converting enzyme (ACE) inhibitor or angiotensin receptor blocker (ARB) therapy. Most studies evaluated BP using office measurements, whereas 1 trial relied on ambulatory monitoring.

Dosing strategies varied by intervention. Baxdrostat was administered at fixed or titrated daily doses of 0.5 mg, 1 mg, 2 mg, or 4 mg; lorundrostat at 12.5 mg, 25 mg, 50 mg, or 100 mg once daily, including titration schedules (eg, 50 mg with subsequent uptitration to 100 mg); and vicadrostat at 3 mg, 10 mg, or 20 mg. Baseline characteristics of the included trials are presented in Table 1, Supplemental Tables 3 and 4.

**Table 1:** Baseline Characteristics of the Included Trials

Study	ADVANCE-HTN^17^ (N = 285)	BrigHTN^32^ (N = 275)	HALO^13^ (N = 249)	LAUNCH-HTN^18^ (N = 1,083)	FigHTN^b^^,^^20^ (N = 195)	Target-HTN^12^ (N = 200)	Tuttle^11^ (N = 288)	BaxHTN^19^ (N = 794)
Year	2025	2023	2023	2025	2025	2023	2024	2025
Trial identification number	NCT05769608	NCT04519658	NCT03996772	NCT06153693	NCT05432167	NCT05001945	NCT05182840	NCT06034743
Country	Multicentric, United States	Multicentric, United States	Multicentric, United States	Multicentric	Multicentric	Multicentric, United States	Multicentric	Multicentric
Follow-up	12 weeks	12 weeks	12 weeks	12 weeks	26 weeks	8 weeks	14 weeks	12 weeks

**Intervention**	**Lorundrostat**	**Baxdrostat**	**Baxdrostat**	**Lorundrostat**	**Baxdrostat**	**Lorundrostat**	**Vicadrostat**	**Baxdrostat**

No. of patients	190 95	206 69	185 64	811 272	129 66	164 36	215 73	530 264
Dose regimen	50 mg daily (n = 94) Dose-adjustment^a^ (n = 96)	0.5 mg (n = 69) 1 mg (n = 70) 2 mg (n = 67)	0.5 mg (n = 63) 1 mg (n = 62) 2 mg (n = 60)	50 mg and then 100 mg (n = 270) 50 mg (n = 541)	High dosing strategy (n = 64) Low dosing strategy (n = 64)	100 mg (n = 61) 50 mg (n = 58) 25 mg (n = 22) 12.5 mg (n = 23)	3 mg (n = 71) 10 mg (n = 72) 20 mg (n = 72)	1 mg (n = 264) 2 mg (n = 266)
Demographics								
Age, y	61.1 59.1	61.7 63.8	60.1 60.5	62.6 61.8	67 66	66.4 62.2	64.5 62.3	60.8 61.9
Male	110 (58) 62 (62)	111 (54) 42 (61)	90 (36) 27 (42)	436 (54) 139 (51)	85 (66) 47 (71)	65 (39) 15 (41)	145 (67) 55 (75)	332 (62.6) 162 (61.4)
Race or ethnic group								
White	74 (39) 41 (43)	140 (67) 51 (74)	135 (73) 46 (72)	556 (68) 177 (65)	75 (58) 38 (58)	107 (65) 17 (65)	127 (59) 44 (60)	333 (63.3) 167 (63.3)
Black	106 (56) 44 (46)	61 (30) 16 (23)	43 (23) 17 (26)	239 (29) 87 (32)	43 (33) 20 (30)	54 (33) 8 (30)	21 (10) 10 (14)	44 (8.3) 15 (5.7)
Other	32 (17) 25 (26)	88 (45) 30 (43)	7 (4) 1 (2)	18 (2) 7 (2)	1 (2) 2 (3)	2 (1) 1 (4)	62 (30) 19 (9)	12 (2.2) 8 (3)
Medical characteristics								
Systolic blood pressure, mm Hg	142.7 141.7	147.53 148.9	146.5 147.9	148.3 148.8	150.9 151.9	141.6 141.6	135.3 133.9	149.4 149.0
Diastolic blood pressure, mm Hg	88.5 90.7	87.9 88.2	NA	87.4 87.1	80.8 81.7	80.2 83.1	77.2 80.2	86.9 85.8
eGFR, mL/min/1.73 m^2^	81.8 79.6	83.1 85.5	NA	91 91	45 44	79.1 82.0	53.2 54.6	85.5 84.1
Diabetes, n (%)	85 (45) 34 (36)	77 (37) 28 (40)	NA	249 (30) 89 (33)	97 (75) 59 (89)	63 (38) 16 (44)	147 (68) 49 (67)	193 (36.4) 110 (41.7)
Potassium, mmol/L	4 4.02	4.13 4.2	NA	NA	4.4 4.3	NA	4.29 4.31	4.2 4.2
BMI, kg/m^2^	31.8 32.2	32.8 32.1	NA	32.93 32.6	31 31.7	31.0 31.9	30.1 30.4	31.3 31.1
Medications								
Thiazide or any diuretic use	190 (100) 95 (100)	206 (100) 69 (100)	NA	779 (96) 259 (95)	54 (42) 31 (47)	105 (64) 23 (64)	NA	527 (99) 264 (100)
ACEi or ARB use	190 (100) 95 (100)	193 (93) 63 (91)	NA	704 (86) 225 (82)	126 (97.6) 62 (93.9)	135 (82) 27 (75)	212 (99) 74 (99)	480 (90) 236 (89)

This table summarizes the key design features and baseline characteristics of the included randomized controlled trials evaluating second-generation aldosterone synthase inhibitors, including sample size, population risk profile, follow-up duration, and dose regimens.

Data are expressed as n (%) or mean ± SD.

ACE = angiotensin-converting enzyme; ARB = angiotensin receptor blocker; BMI = body-mass index; eGFR = estimated glomerular filtration rate; NA = not reported.

a Starting dose of 50 mg daily, with an increase to 100 mg daily if systolic blood pressure was 130 mm Hg or higher after 4 weeks.

b High dosing (0.5 mg uptitrated to 1 mg); Low dosing (2 mg uptitrated to 4 mg).

### Efficacy outcomes

**Figure 2:**
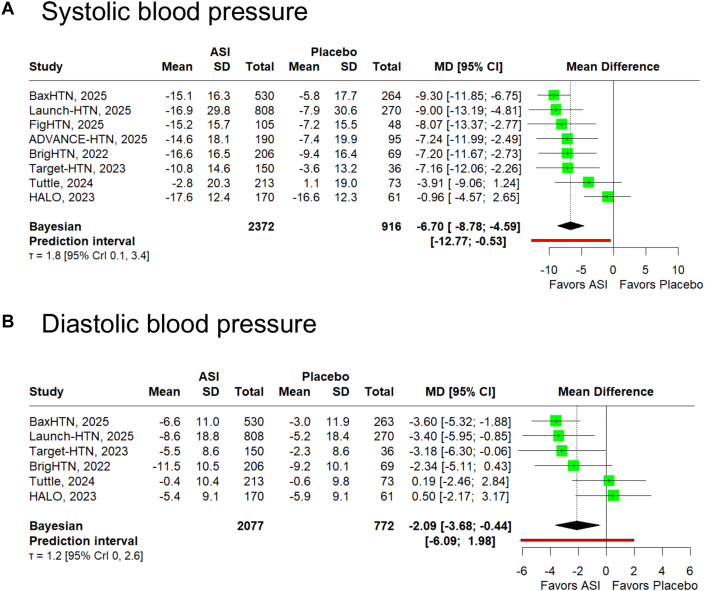
**Bayesian Meta-Analysis of the Effects of Aldosterone Synthase Inhibitor vs Placebo on Blood Pressure** Forest plots show change from baseline in (A) systolic and (B) diastolic blood pressure. ASI = aldosterone synthase inhibitors; CrI = credible interval; MD = mean difference.

Compared with placebo, ASIs reduced the mean SBP (MD: −6.70 mm Hg; 95% CrI: −8.78, −4.59; τ^2^ 3.24) and DBP (MD: −2.09 mm Hg; 95% CrI: −3.68, −0.44; τ^2^ 1.44), with posterior probabilities of 94.6% and 54.1% for achieving clinically meaningful reductions (−5 mm Hg for SBP and −2 mm Hg for DBP), respectively (Figure 2). The posterior predictive probabilities of clinically meaningful benefit in new comparable studies were 80.3% for SBP and 54.2% for DBP. The proportion of patients achieving SBP target was higher with ASI than placebo (OR: 2.34; CrI: 1.61, 3.37; τ^2^ 0.07). When stratified by dose, the reduction in the mean SBP was −2.24 mm Hg (CrI: −5.37, 0.88; τ^2^ 0.81) for low-dose ASIs, −6.46 mm Hg (CrI: −8.93, −3.94; τ^2^ 4.0) with intermediate doses, and −7.90 mm Hg (CrI: −10.59, −5.12; τ^2^ 1.96) with high doses (Supplemental Figures 1A to 1D).

### Safety outcomes

Treatment with ASI reduced the odds of hypertensive urgency (OR: 0.36; CrI: 0.13, 0.9; τ^2^ 0.12). However, hyperkalemia (OR: 7.1; CrI: 3.56, 15.2; τ^2^ 0.23), severe hyperkalemia (≥6.0 mmol/L) (OR: 12.55; CrI: 3.52, 61.9; τ^2^ 0.1), hyponatremia (OR: 2.6; CrI: 1.25, 5.98; τ^2^ 0.1), and hypotension (OR: 3.28; CrI: 1.43, 8.16; τ^2^ 0.1) were more frequent with ASIs. There were no differences in serious adverse events (OR: 1.4; CrI: 0.77, 2.85; τ^2^ 0.1), drug-related adverse events (OR: 8.33; CrI: 0.67, 232.75; τ^2^ 0.1), all-cause mortality (OR: 0.45; CrI: 0.06, 3.23; τ^2^ 0.1), adrenal insufficiency (OR: 0.5; CrI: 0.1, 3.1; τ^2^ 0.29), or any arrhythmia (OR: 1.07; CrI: 0.3, 4.8; τ^2^ 0.14) between groups (Table 2, Supplemental Figures 2A to 2J). Main results are summarized in the Central Illustration.

**Table 2:** Safety Outcomes

	Number of Studies	ASI Events/Patients (%)	Placebo Events/Patients (%)	OR (CrI)	Heterogeneity (tau)
Any serious adverse event	8	69/2,450 (2.8)	22/935 (2.3)	1.4 (0.77-2.85)	0.31 (0-0.81)
Drug-related serious adverse events	5	5/1,494 (0.3)	0/534 (0)	8.33 (0.67-232.75)	0.33 (0-0.96)
Hyperkalemia	8	207/2,415 (8.5)	15/931 (1.6)	7.1 (3.56-15.2)	0.48 (0-1.03)
Severe hyperkalemia (K ≥6.0 mmol/L)	5	41/1891 (2.1)	2/732 (0.27)	12.55 (3.52-61.9)	0.31 (0-0.94)
Hyponatremia	4	96/1,656 (5.7)	16/693 (2.3)	2.6 (1.25-5.98)	0.3 (0-0.83)
Hypotension	7	57/2,238 (2.5)	8/871 (0.9)	3.28 (1.43-8.16)	0.3 (0-0.79)
Hypertensive urgency	4	16/1,311 (1.2)	16/493 (3.2)	0.36 (0.13-0.9)	0.36 (0-0.85)
All-cause mortality	8	2/2,423 (0.1)	2/935 (0.2)	0.45 (0.06-3.23)	0.33 (0-1.01)
Adrenal insufficiency	5	4/1905 (0.2)	3/738 (0.4)	0.5 (0.1-3.1)	0.54 (0-1.26)
Any arrhythmia	4	8/707 (1.1)	4/291 (1.3)	1.07 (0.3-4.8)	0.34 (0-0.97)

ASI = aldosterone synthase inhibitors; CrI = credible interval.

**Central Illustration:**
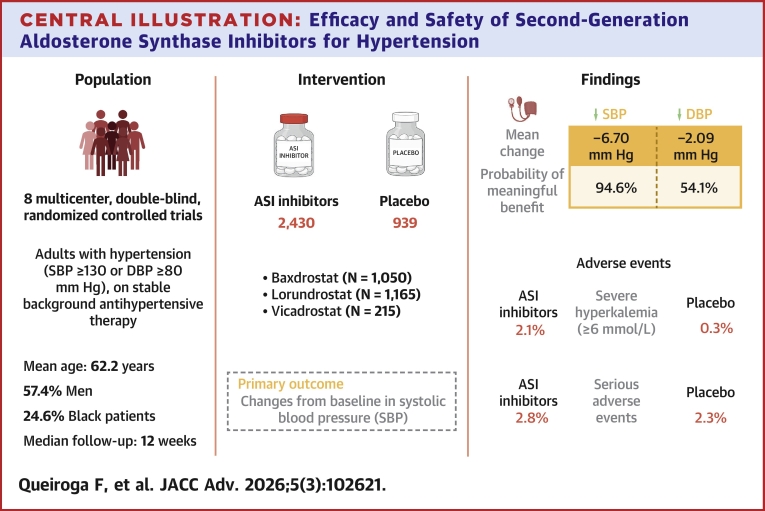
**Efficacy and Safety of Second-Generation Aldosterone Synthase Inhibitors for Hypertension** Created in BioRender. Firmino F (2025), https://BioRender.com/2i008vu. ASI = aldosterone synthase inhibitors; DBP = diastolic blood pressure; SBP = systolic blood pressure.

### Subgroup and sensitivity analysis

**Figure 3:**
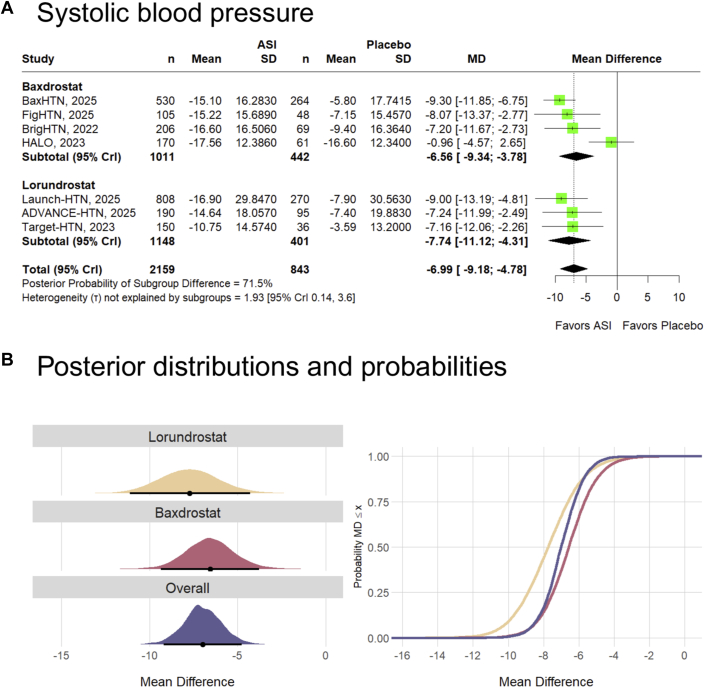
**Subgroup Analysis by Drug Type (Baxdrostat vs Lorundrostat)** (A) Forest plot; (B) Posterior distributions and probabilities for systolic blood pressure. Point estimates (black solid-circle data markers) depict the median, and interval bars represent the 95% credible (highest-density) intervals. Cumulative posterior probabilities correspond to the probability that the MD is lower than or equal to the effect size on the x-axis (X). Abbreviations as in Figure 2.

When stratified by drug type, the pooled MD for SBP was −6.56 mm Hg (95% CrI: −9.34, −3.78) for baxdrostat and −7.74 mm Hg (95% CrI: −11.12, −4.31) for lorundrostat (Figure 3). The posterior probability that treatment effect differed by drug type was 71.5%. In subgroup analysis, the likelihood of achieving a clinically meaningful reduction in SBP was 87.5% with baxdrostat and 94.3% with lorundrostat.

For other outcomes, the posterior probability of subgroup differences was 75.0% for DBP and 69.4% for achieving SBP target (Supplemental Figures 3A and 3B).

Leave-one-out analysis showed that exclusion of HALO trial modestly increased the pooled SBP effect (−7.9 mm Hg). For BP target, results were consistent, but for DBP estimates, varied modestly depending on the study removed (Supplemental Figures 4A to 4C).

Sensitivity analysis showed that overall results for SBP were not heavily influenced by different priors. Posterior predictive distribution was not dependent on the choice of prior for the between-study heterogeneity parameter. In the sensitivity analysis using a skeptical weakly informative prior centered at zero for the overall effect, the reduction in SBP was −6.58 mmHg (CrI: −8.95, −4.16), with an 99.9% probability of any benefit and an 91.1% predictive probability of any benefit in new comparable studies (Supplemental Figures 5A to 5D).

### Dose-response analysis

For baxdrostat, increasing doses up to 2 mg were associated with progressively greater reductions in SBP, with the probability of achieving a ≥5-mm Hg reduction rising from 0% at 0.5 mg to 98% at 2.0 mg. Although the probability of increased risk for serious adverse events increased numerically with dose, no consistent dose-response relationship was found for hyperkalemia or serious adverse events (Supplemental Tables 5A to 5C, Supplemental Figures 6A to 6C).

### Quality assessment

Six trials were rated as low risk of bias, and 2 as some concerns due to missing outcome data and selective reporting. Funnel plot inspection suggested mild asymmetry, raising the possibility of publication bias. However, the Egger test does not indicate the presence of funnel plot asymmetry (*P* = 0.53) (Supplemental Table 6, Supplemental Figures 7 and 8).

## Discussion

This systematic review and Bayesian meta-analysis of 8 placebo-controlled RCTs including 3,369 patients on optimized background antihypertensive therapy showed that second-generation ASIs produced clinically meaningful reductions in systolic and diastolic BPs. ASI therapy also increased the likelihood of achieving a target SBP and reduced the incidence of hypertensive urgency. A dose-response effect was observed, with high-dose ASIs yielding a mean SBP reduction of −7.9 mm Hg. ASIs did cause hyperkalemia, hyponatremia, and hypotension, although adverse events were overall infrequent.

Although MRAs remain a central therapy for resistant hypertension, the advent of ASIs introduces a distinct upstream approach to aldosterone targeting.^6^^,^^10^ By reducing aldosterone production rather than blocking its receptor, ASIs may address residual aldosterone activity and the rebound phenomenon observed with ACE inhibitors, ARBs, and MRAs, and may also have a different hormonal side-effect profile.^7^^,^^8^^,^^10^^,^^14^ This mechanism provides a plausible basis for the BP reductions observed in our analysis. Whether these mechanistic differences translate into improved cardiovascular outcomes remains uncertain.^33^

BrigHTN,^32^ a phase II trial of baxdrostat in resistant hypertension, stopped early for overwhelming efficacy, and subsequent trials with both baxdrostat, lorundrostat, and vicadrostat also demonstrated meaningful reductions in SBP.^11^^,^^18^^,^^19^ Notably, higher doses of second-generation ASIs achieved BP reductions comparable to the −8.70 mm Hg observed with spironolactone in the PATHWAY-2^34^ trial. In this trial, 6 of 285 patients (2%) treated with spironolactone experienced hyperkalemia, whereas in our pooled analysis of second-generation ASIs, the incidence was 41 of 1,891 patients (2%), suggesting a similar overall risk profile. For hypotension, 57 of 2,238 patients (2.5%) treated with ASIs experienced this event in our pooled analysis. In a post hoc analysis including data from FIDELITY-TRH and AMBER, hypotension occurred in 5 of 316 patients (1.6%) with finerenone and 9 of 146 patients (6%) with spironolactone.^35^

ASIs' antihypertensive effect varied across trials, and baseline study characteristics may have influenced these results. The modest efficacy of vicadrostat observed in our analysis may reflect lower intrinsic potency and the inclusion of participants with lower baseline BP and renal function. The HALO trial was a notable outlier in the baxdrostat group, reporting no between-group SBP difference; however, poor adherence at certain sites likely diluted treatment effects, as post hoc analysis revealed subtherapeutic drug levels in participants.^36^

Regarding safety, hyperkalemia, hyponatremia, and hypotension were the most frequent adverse events. Importantly, adrenal insufficiency was rare, aligning with the improved selectivity anticipated for second-generation compounds compared with prior-generation ASIs.^10^^,^^14^^,^^37^ In our pooled analysis, the incidence of adrenal insufficiency was very low (4 of 1,905 patients [0.2%]), underscoring the enhanced safety profile. However, ASIs were associated with an increased risk of severe hyperkalemia (41 of 1,891 patients [2.2%]) compared with placebo (2 of 732 [0.27%]), which was likely influenced by the high rate of concomitant ACE inhibitors or ARBs in several included studies. Because this reflects real-world practice in resistant hypertension, careful potassium monitoring will remain essential in clinical use.

Recent studies have begun to explore the use of ASIs in combination with SGLT2 inhibitors.^11^ In a phase-2 trial in CKD, adding empagliflozin to an ASI provided additive reductions in albuminuria and attenuated the rise in serum potassium seen with higher ASIs doses.^11^ This suggests that SGLT2 inhibitors contribute to additional BP lowering and may also mitigate hyperkalemia. Phase-2 and -3 programs are now evaluating second-generation ASIs combined with dapagliflozin and empagliflozin which will provide more definitive evidence in the context of resistant hypertension.^38^

Unlike prior meta-analyses^15^^,^^16^ we applied a Bayesian approach, which allowed the estimation of 95% CrI and clinically relevant posterior probabilities. Specifically, we calculated the probability of achieving a clinically meaningful SBP reduction (−5 mm Hg), a threshold associated with lower rates of cardiovascular events and end-organ damage.^24^^,^^25^ Our approach provides a more intuitive interpretation of the evidence and may better inform clinical decision-making than conventional frequentist methods.^39^ Although previous studies have shown that ASIs reduce SBP, our Bayesian approach reinforces this evidence by demonstrating a high probability of a clinically significant benefit. Furthermore, posterior predictive analyses incorporating hypothetical future data yielded consistent results, strengthening the robustness and applicability of our findings. In addition, by including only newer RCTs of second-generation ASIs not available in previous syntheses, our analysis nearly doubles the total randomized population, providing a more up-to-date estimate of ASIs efficacy and safety.

Future studies should directly compare second-generation ASIs with MRAs in patients with resistant hypertension, CKD, and heart failure, evaluating long-term BP control, cardio-renal outcomes, and safety. Strategies to reduce hyperkalemia, including use of potassium binders, or combination therapy warrant investigation.

### Study Limitations

Our study has several limitations. First, heterogeneity across drugs, dosages, patient populations, outcome measurements, and background antihypertensive therapies may have influenced the results; however, we performed sensitivity analyses to address these factors. Second, most trials had relatively short follow-up, restricting the ability to evaluate long-term cardiovascular and safety outcomes. Third, most participants had preserved renal function, which may have underestimated the risk of adverse events, such as hyperkalemia. Fourth, the absence of direct head-to-head comparisons with active agents precludes definitive conclusions regarding relative efficacy. In addition, most studies relied on office-based BP measurements, although ADVANCE-HTN and a BaxHTN subgroup incorporated 24-hour ambulatory BP monitoring, which yielded results consistent with conventional measurements in our analysis. Fifth, this analysis is based on the study-level data rather than individual patient data, which should be considered when interpreting the results. Sixth, funnel plot assessment suggested possible small-study effects that could inflate our pooled estimates, and although the Egger test was not significant, publication bias cannot be excluded given the small number of studies. Lastly, we were unable to analyze the impact of treatment adherence on outcomes given the lack of reporting.

## Conclusions

Second-generation ASIs had a high likelihood of a clinically significant reduction in SBP and DBP compared with placebo. ASIs increased the risk of hyperkalemia, hyponatremia, and hypotension, although serious adverse events were overall infrequent.

Perspectives **COMPETENCY IN MEDICAL KNOWLEDGE:** Second-generation ASIs significantly reduce BP in patients with hypertension with an acceptable safety profile. These agents may offer an alternative treatment option for patients who cannot tolerate traditional RAAS blockade.

**TRANSLATIONAL OUTLOOK:** Additional studies are needed to determine whether these agents offer cardiovascular protection comparable to MRAs and whether their antihypertensive effects are durable across broader populations. Head-to-head trials, long-term safety evaluations, and assessments of metabolic and steroidogenic effects remain priorities. Research should also address cost-effectiveness and practical integration into hypertension care.

## Funding support and author disclosures

Dr Bhatt discloses he serves on the Advisory Board of Angiowave, Antlia Bioscience, 10.13039/100004326Bayer, Boehringer Ingelheim, CellProthera, Cereno Scientific, E-Star Biotech, High Enroll, Janssen, Level Ex, McKinsey, Medscape Cardiology, 10.13039/100004334Merck, NirvaMed, Novo Nordisk, Repair Biotechnologies, Stasys, SandboxAQ (stock options), Tourmaline Bio, and Viatris; he is a Board of Director member for the American Heart Association New York City, Angiowave (stock options), Bristol Myers Squibb (stock), DRS.LINQ (stock options), and High Enroll (stock); he is a consultant for Alnylam, Altimmune, Broadview Ventures, Corcept Therapeutics, Corsera, GlaxoSmithKline, Hims, SERB, SFJ, Summa Therapeutics, and Worldwide Clinical Trials; he serves on the Data Monitoring Committees for Acesion Pharma, Assistance Publique-Hôpitaux de Paris, Baim Institute for Clinical Research, Boston Scientific (Chair, PEITHO trial), Cleveland Clinic, Contego Medical (Chair, PERFORMANCE 2), Duke Clinical Research Institute, Mayo Clinic, Mount Sinai School of Medicine (for the ABILITY-DM trial, funded by Concept Medical; for ALLAY-HF, funded by Alleviant Medical), Novartis, Population Health Research Institute, and Rutgers University (for the NIH-funded MINT Trial); he receives honoraria from American College of Cardiology (Senior Associate Editor, Clinical Trials and News, ACC.org; Chair, ACC Accreditation Oversight Committee), Arnold and Porter law firm (work related to Sanofi/Bristol-Myers Squibb clopidogrel litigation), Baim Institute for Clinical Research (AEGIS-II executive committee funded by CSL Behring), Belvoir Publications (Editor in Chief, Harvard Heart Letter), Canadian Medical and Surgical Knowledge Translation Research Group (clinical trial steering committees), CSL Behring (AHA lecture), Duke Clinical Research Institute, Engage Health Media, HMP Global (Editor in Chief, Journal of Invasive Cardiology), Medtelligence/ReachMD (CME steering committees), MJH Life Sciences, Oakstone CME (Course Director, Comprehensive Review of Interventional Cardiology), Philips (Becker's Webinar on AI), Population Health Research Institute, WebMD (CME steering committees), and Wiley (steering committee); he holds other roles in Clinical Cardiology (Deputy Editor, unpaid), Progress in Cardiovascular Diseases (Deputy Editor, unpaid), and Added Health (Editorial Board; stock options); he has a patent for Sotagliflozin (named on a patent for sotagliflozin assigned to Brigham and Women's Hospital who assigned to Lexicon; neither I nor Brigham and Women's Hospital receive any income from this patent); he receives research funding from 10.13039/100000046Abbott, Acesion Pharma, Afimmune, 10.13039/100006400Alnylam, Amarin, 10.13039/100002429Amgen, 10.13039/100004325AstraZeneca, 10.13039/100007330Atricure, 10.13039/100004326Bayer, 10.13039/100001003Boehringer Ingelheim, Boston Scientific, CellProthera, Cereno Scientific, Chiesi, Cleerly, CSL Behring, Faraday Pharmaceuticals, Fractyl, Idorsia, Janssen, Javelin, Lexicon, Lilly, Medtronic, Merck, MiRUS, Moderna, Novartis, 10.13039/501100004191Novo Nordisk, Pfizer, PhaseBio, Regeneron, Reid Hoffman Foundation, Roche, Sanofi, Stasys, and 89Bio; he receives royalties from Elsevier (Editor, Braunwald’s Heart Disease); and he is a site co-investigator for Cleerly. All other authors have reported that they have no relationships relevant to the contents of this paper to disclose.
