# Supplementary material for: Second-Generation Aldosterone Synthase Inhibitors for Hypertension: A Bayesian Meta-Analysis of Randomized Trials
Source: JACC Adv. 2026 Feb 27;5(3):102621. doi: 10.1016/j.jacadv.2026.102621 (PMC13100762; doi:10.1016/j.jacadv.2026.102621)
Supplement: Supplemental material [file mmc1.docx]

**SUPPLEMENTAL APPENDIX**

**Second-Generation Aldosterone Synthase Inhibitors for Hypertension: A Bayesian Meta-Analysis of Randomized Trials**

**Table of Contents**

[Supplemental Methods. Study-Level Bayesian Meta-Analysis 3](#_Toc219920694)

[Supplemental Table 1. Prisma checklist 5](#_Toc219920695)

[Supplemental Table 2. Search strategy 12](#_Toc219920696)

[Supplemental Table 3. Inclusion and exclusion criteria by study 13](#_Toc219920697)

[Supplemental Table 4. Characteristics of included studies 17](#_Toc219920698)

[Supplemental Table 5. Dose-Response Analyses 21](#_Toc219920699)

[Supplemental Table 6. Risk of bias assessment of RCTs with RoB-2 tool 23](#_Toc219920700)

[Supplemental Figure 1. Efficacy outcomes 24](#_Toc219920701)

[Supplemental Figure 2. Safety outcomes 26](#_Toc219920702)

[Supplemental Figure 4: Leave-one-out analysis 37](#_Toc219920703)

[Supplemental Figure 5. Sensitivity analysis for systolic blood pressure 39](#_Toc219920704)

[Supplemental Figure 6. Dose-response analysis 41](#_Toc219920707)

[Supplemental Figure 7. Funnel plot for systolic blood pressure 44](#_Toc219920708)

[Supplemental Figure 8. Egger’s test for systolic blood pressure 44](#_Toc219920709)

# Supplemental Methods. Study-Level Bayesian Meta-Analysis

**1.Efficacy outcomes**

For each outcome, we fitted a Bayesian random-effects meta-analysis model.^1,2^ This model consists of observed mean log mean differences from each study. We assume these effect sizes are normally distributed around the true study-specific means along with a known sampling variance. We also assume that these study-specific means are drawn from another normal distribution where “mu” is the average effect (mean), and “tau” is the between-study heterogeneity (standard deviation).

**Priors**

**Mean difference for systolic blood pressure**

– Primary analysis, non-informative for “mu”, weakly informative for “tau”

– Sensitivity analyses, vague for “mu”, vague for "tau”

Because these are Bayesian models, we need to specify prior distributions for all parameters. In the primary analysis, we applied a non-informative prior for “mu”, where all relevant values are equally likely, corresponding to no prior knowledge for the average effect: Normal (0, 510^2^), 95% of the probability density ranging from -1000 to 1000 mean difference, as depicted below:


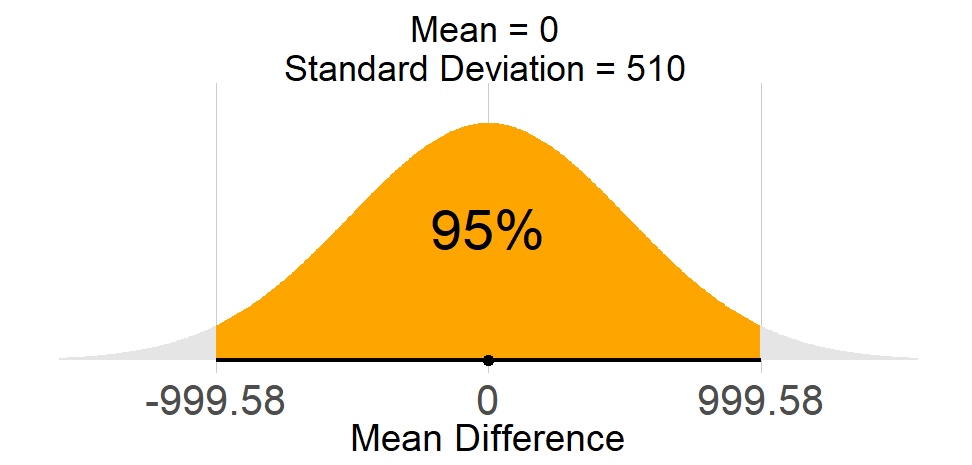


For the between-study heterogeneity parameter (“tau”), we used a weakly informative prior, half-normal (1.67), 95% of the probability density ranging from 1.1 to 3.3 influenced by recommendations from Rover et al. 2021 and Spiegelhalter et al., 2003:


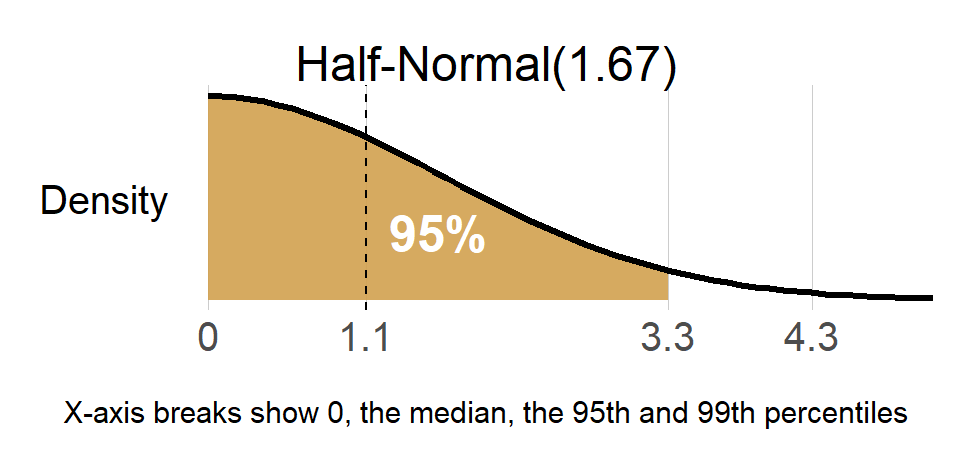


To derive this prior, we first identified a "surprisingly large" effect size for this outcome, **6.42 mmHg**, based on **median systolic blood pressure reduction of the placebo group in previous meta-analyses**. We calculated the scale parameter such that the 97.5th percentile of a normal distribution (mean = 0, standard deviation = “tau_e”) equals this extreme value, and “tau_e” is the 95th percentile of the half-normal prior. The resulting prior has a 97.5th percentile of **6.42**, implying 95% of true study effects range from **-6.42** to **6.42** mean difference.


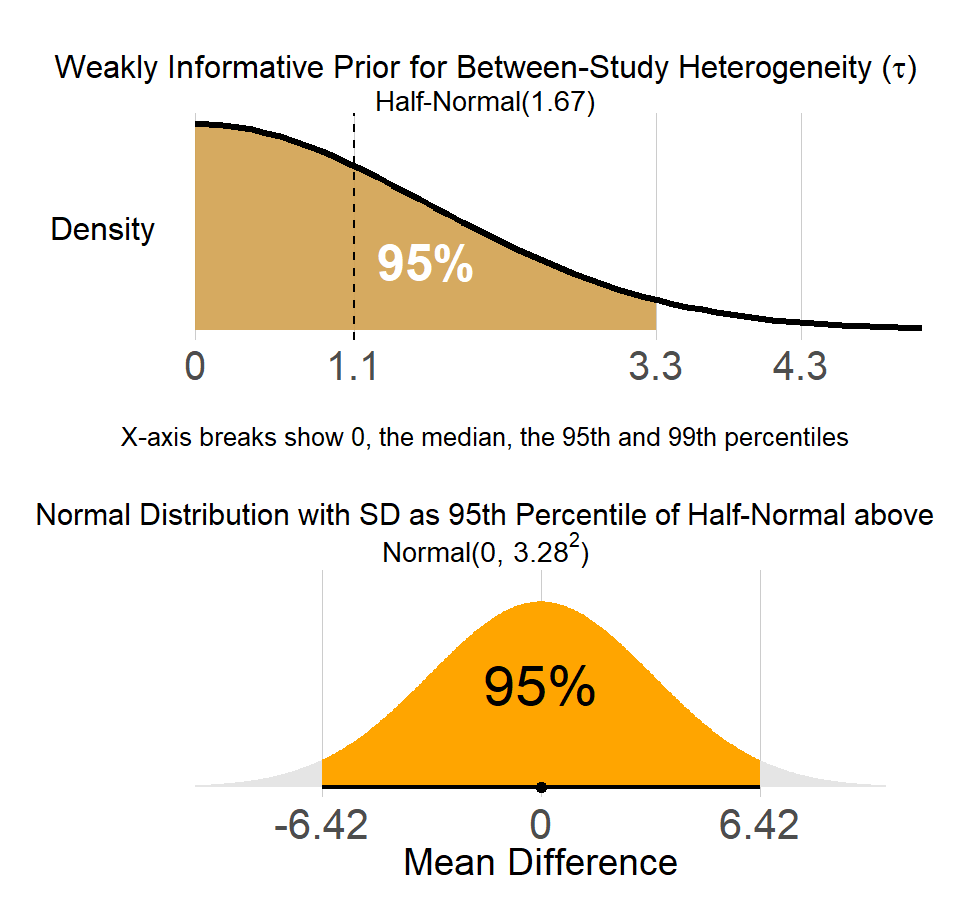


This plot illustrates the relationship between the half-normal prior for and a normal distribution of true study effects, where the standard deviation is half-normal's 95th percentile. This visualization clarifies how the prior constrains heterogeneity to plausible ranges, as recommended by Röver et al.^3^

For further technical details of the rationale for prior elicitation, reference to Sections 5.7.3 and 8.4 in Spiegelhalter et al.^4^

**Summary of priors for efficacy outcomes**

| **Outcome** | **Effect Size** | **Prior Type for Mu^a^** | **Mu Prior Distribution^a,b^** | **Prior Type for Tau^c^** | **Tau Prior Distribution^d^** |
| --- | --- | --- | --- | --- | --- |
| Systolic blood pressure | Mean Difference | Vague | Normal (0, 510^2^) | Weakly-informative | Half-Normal (1.67) |
| Diastolic blood pressure | Mean Difference | Vague | Normal (0, 510^2^) | Weakly-informative | Half-Normal (0.97) |
| Systolic blood  pressure target | Risk Ratio | Vague | Normal (0, 2.7) | Informative | Log-Normal (-0.885, 0.76) |

^a^Mu = Overall effect parameter

^b^Normal (mean, standard deviation)

^c^Tau = Between-study standard deviation parameter

^d^Log-normal (log-mean, standard deviation); Half-normal(scale)

**2. Safety outcomes**

Because safety events were infrequent, we analyzed these endpoints using a binomial–normal hierarchical model in MetaStan, which is well suited for sparse binary data and avoids the need for continuity corrections. In this framework, study-specific log-odds ratios are assumed to arise from a normal distribution with an overall treatment effect “theta” and a between-study heterogeneity parameter “tau”.

**Priors**

-Treatment effect “theta”

We applied MetaStan’s recommended weakly informative prior for rare-event meta-analysis.^5^ This is defined through an upper bound of delta = 250 on the odds-ratio scale, which corresponds to a Normal(0, 2.82²) prior on the log-odds ratio. This prior allows for a wide range of plausible treatment effects without permitting extreme or unstable values.

Between-study heterogeneity “tau”

A weakly informative Half-Normal (0, 0.5) prior was used to regularize the heterogeneity estimate while still allowing for meaningful between-study variation.

**2.1. Summary of priors for safety outcomes**

| **Outcome** | **Effect Size** | **Prior Type for Theta^a^** | **Theta Prior Distribution^a,b^** | **Prior Type for Tau^c^** | **Tau Prior Distribution^d^** |
| --- | --- | --- | --- | --- | --- |
| Safety outcomes* | Odds Ratio | Weakly informative | Normal (0, 2.82²) | Weakly informative | Log-Normal (0, 0.5) |

^a^Theta = Treatment effect parameter

^b^Normal (mean, standard deviation)

^c^Tau = Between-study standard deviation parameter

^d^Log-normal (log-mean, standard deviation); Half-normal(scale)

^*^Include serious adverse events, drug-related adverse events, hyperkalemia, severe hyperkalemia, hypertensive urgency, hyponatremia, hypotension, adrenal insufficiency, and any arrythmia.

**2.2. Convergence criteria and diagnostic output**

| **Outcome** | **Maximum R-hat** | **Minimum effective sample size (ESS)** | **Overall assessment** |
| --- | --- | --- | --- |
| **Systolic blood pressure** | 1.001 | 13,000 | Converged |
| **Hyperkalemia** | 1.004 | 2,200 | Converged |
| **Serious adverse events** | 1.006 | 380 | Converged |

**3. Dose -response**

**3.1. Summary of priors**

| **Outcome** | **Effect Size** | **Prior Type for Pooled Spline Coeficients** | **Pooled Spline Coeficient Prior Distribution** | **Prior Type for Between-Study Heterogeneity** | **Between-Study Heterogeneity Prior Distribution** |
| --- | --- | --- | --- | --- | --- |
| Systolic blood pressure | Mean Difference | Vague | Normal (mean = 0, precision = 2) | Weakly-informative | sd1: Normal (0, precision = 1) truncated to (0,1); sd2: Normal (0, precision = 1) truncated to (0,1); rho: Uniform (-1, 1) |
| Hyperkalemia and serious adverse events | Risk Ratio | Vague | Normal (mean = 0, precision = 2) | Weakly-informative | sd1: Uniform (0, 2); sd2: Uniform (0, 2); rho: Uniform (-0.95, 0.95) |

^a^sd1 and sd2 = between-study standard deviations for the two spline coefficients, capturing heterogeneity in the dose–response relationship across studies

^b^Rho = correlation between the two random-effects components (sd1 and sd2), allowing heterogeneity in the spline parameters to be correlated across studies

# Supplemental Table 1. Prisma checklist

| **Section and Topic** | **Item #** | **Checklist item** | **Location where item is reported** |
| --- | --- | --- | --- |
| **TITLE** | | |  |
| Title | 1 | Identify the report as a systematic review. | Page 1. |
| **ABSTRACT** | | |  |
| Abstract | 2 | See the PRISMA 2020 for Abstracts checklist. | Page 1. |
| **INTRODUCTION** | | |  |
| Rationale | 3 | Describe the rationale for the review in the context of existing knowledge. | Page 2. |
| Objectives | 4 | Provide an explicit statement of the objective(s) or question(s) the review addresses. | Page 2. |
| **METHODS** | | |  |
| Eligibility criteria | 5 | Specify the inclusion and exclusion criteria for the review and how studies were grouped for the syntheses. | Page 2. |
| Information sources | 6 | Specify all databases, registers, websites, organizations, reference lists and other sources searched or consulted to identify studies. Specify the date when each source was last searched or consulted. | Page 2. |
| Search strategy | 7 | Present the full search strategies for all databases, registers and websites, including any filters and limits used. | Page 2 and Supplemental Table 2. |
| Selection process | 8 | Specify the methods used to decide whether a study met the inclusion criteria of the review, including how many reviewers screened each record and each report retrieved, whether they worked independently, and if applicable, details of automation tools used in the process. | Page 2. |
| Data collection process | 9 | Specify the methods used to collect data from reports, including how many reviewers collected data from each report, whether they worked independently, any processes for obtaining or confirming data from study investigators, and if applicable, details of automation tools used in the process. | NA. |
| Data items | 10a | List and define all outcomes for which data were sought. Specify whether all results that were compatible with each outcome domain in each study were sought (e.g. for all measures, time points, analyses), and if not, the methods used to decide which results to collect. | Pages 2 and 3. |
|  | 10b | List and define all other variables for which data were sought (e.g. participant and intervention characteristics, funding sources). Describe any assumptions made about any missing or unclear information. | NA. |
| Study risk of bias assessment | 11 | Specify the methods used to assess risk of bias in the included studies, including details of the tool(s) used, how many reviewers assessed each study and whether they worked independently, and if applicable, details of automation tools used in the process. | Page 3. |
| Effect measures | 12 | Specify for each outcome the effect measure(s) (e.g. risk ratio, mean difference) used in the synthesis or presentation of results. | Page 3. |
| Synthesis methods | 13a | Describe the processes used to decide which studies were eligible for each synthesis (e.g. tabulating the study intervention characteristics and comparing against the planned groups for each synthesis (item #5)). | Pages 2. |
|  | 13b | Describe any methods required to prepare the data for presentation or synthesis, such as handling of missing summary statistics, or data conversions. | Page 3. |
|  | 13c | Describe any methods used to tabulate or visually display results of individual studies and syntheses. | Page 3. |
|  | 13d | Describe any methods used to synthesize results and provide a rationale for the choice(s). If meta-analysis was performed, describe the model(s), method(s) to identify the presence and extent of statistical heterogeneity, and software package(s) used. | Page 3. |
|  | 13e | Describe any methods used to explore possible causes of heterogeneity among study results (e.g. subgroup analysis, meta-regression). | Page 3. |
|  | 13f | Describe any sensitivity analyses conducted to assess robustness of the synthesized results. | Page 3. |
| Reporting bias assessment | 14 | Describe any methods used to assess risk of bias due to missing results in a synthesis (arising from reporting biases). | Page 3. |
| Certainty assessment | 15 | Describe any methods used to assess certainty (or confidence) in the body of evidence for an outcome. | NA. |
| **RESULTS** | | |  |
| Study selection | 16a | Describe the results of the search and selection process, from the number of records identified in the search to the number of studies included in the review, ideally using a flow diagram. | Pages 3 and 4. |
|  | 16b | Cite studies that might appear to meet the inclusion criteria, but which were excluded, and explain why they were excluded. | Figure 1. |
| Study characteristics | 17 | Cite each included study and present its characteristics. | Pages 3 and 4; Table 1; Figure 1; Supplemental Tables S3 and S4. |
| Risk of bias in studies | 18 | Present assessments of risk of bias for each included study. | Page 7; Supplemental Table 6. |
| Results of individual studies | 19 | For all outcomes, present, for each study: (a) summary statistics for each group (where appropriate) and (b) an effect estimate and its precision (e.g. confidence/credible interval), ideally using structured tables or plots. | Pages 4, 5, and 6; Figures 2 and 3; Supplemental Figures S1 and S2 |
| Results of syntheses | 20a | For each synthesis, briefly summarise the characteristics and risk of bias among contributing studies. | Page 7; Supplemental Table 6. |
|  | 20b | Present results of all statistical syntheses conducted. If meta-analysis was done, present for each the summary estimate and its precision (e.g. confidence/credible interval) and measures of statistical heterogeneity. If comparing groups, describe the direction of the effect. | Pages 4, 5, and 6; Figures 2 and 3; Table 2; Supplemental Figures S1, S2, S5 |
|  | 20c | Present results of all investigations of possible causes of heterogeneity among study results. | Pages 6 and 7; Supplemental Figures S4 and S5. |
|  | 20d | Present results of all sensitivity analyses conducted to assess the robustness of the synthesized results. | Pages 6 and 7; Supplemental Figures S4 and S5. |
| Reporting biases | 21 | Present assessments of risk of bias due to missing results (arising from reporting biases) for each synthesis assessed. | Page 7; Supplemental Figure 7 and S8. |
| Certainty of evidence | 22 | Present assessments of certainty (or confidence) in the body of evidence for each outcome assessed. | NA. |
| **DISCUSSION** | | |  |
| Discussion | 23a | Provide a general interpretation of the results in the context of other evidence. | Pages 7, 8, 9, and 10. |
|  | 23b | Discuss any limitations of the evidence included in the review. | Page 10. |
|  | 23c | Discuss any limitations of the review processes used. | Page 10. |
|  | 23d | Discuss implications of the results for practice, policy, and future research. | Page 10 and 11. |
| **OTHER INFORMATION** | | |  |
| Registration and protocol | 24a | Provide registration information for the review, including register name and registration number, or state that the review was not registered. | PROSPERO; CRD420251132306. |
|  | 24b | Indicate where the review protocol can be accessed, or state that a protocol was not prepared. | https://www.crd.york.ac.uk/PROSPERO/view/CRD420251132306 |
|  | 24c | Describe and explain any amendments to information provided at registration or in the protocol. | Replace original model with a Bayesian binominal-normal hierarchical model for rare event data. |
| Support | 25 | Describe sources of financial or non-financial support for the review, and the role of the funders or sponsors in the review. | NA. |
| Competing interests | 26 | Declare any competing interests of review authors. | Pages 10. |
| Availability of data, code and other materials | 27 | Report which of the following are publicly available and where they can be found template data collection forms; data extracted from included studies; data used for all analyses; analytic code; any other materials used in the review. | NA. |

***Abbreviations:*** NA, not available.

# Supplemental Table 2. Search strategy

| **PubMed** | (lorundrostat OR "MLS-101" OR baxdrostat OR "CIN-107" OR vicadrostat OR "BI 690517" OR "aldosterone synthase inhibitor*") |
| --- | --- |
| **Embase** | 'lorundrostat'/exp OR lorundrostat OR 'mls-101'/exp OR 'mls-101' OR 'baxdrostat'/exp OR baxdrostat OR 'cin-107'/exp OR 'cin-107' OR 'vicadrostat'/exp OR vicadrostat OR 'bi 690517' OR 'aldosterone synthase inhibitor*' |
| **Cochrane** | (lorundrostat OR "MLS-101" OR baxdrostat OR "CIN-107" OR vicadrostat OR "BI 690517" OR "aldosterone synthase inhibitor*") |

# Supplemental Table 3. Inclusion and exclusion criteria by study

| **Study** | **Inclusion criteria** | **Exclusion criteria** |
| --- | --- | --- |
| **ADVANCE-HTN, 2025** | Screening AOBP SBP 140–180 mm Hg or DBP 65–110 mm Hg (or DBP 90–110 mm Hg); Randomization ABPM SBP 130–180 mm Hg or DBP >80 mm Hg; 2–5 stable antihypertensive drugs ≥1 month; hypertension ≥6 months; serum cortisol 3–22 μg/dL (AM); BMI 18–40 kg/m²; contraception if fertile; compliance with visits. | Pregnant/breastfeeding; hypersensitivity to lorundrostat; prohibited medications or recent investigational study; allergy/intolerance to major antihypertensives; eGFR <45 mL/min/1.73 m²; serum potassium >5.0 mmol/L or sodium <135 mmol/L; secondary hypertension (except sleep apnea or primary aldosteronism if MRA discontinued per run-in); recent HF, MI, stroke, TIA; diabetes with HbA1c >9% or severe hypoglycemia/DKA; major surgery planned; recent malignancy; substance abuse; other PI discretion. |
| **BrigHTN, 2023** | Adult ≥18 years; on stable regimen of ≥3 antihypertensive agents (including non–potassium-sparing diuretic) for ≥2 weeks; mean seated BP ≥130/80 mm Hg (after MRA discontinuation if applicable); agrees to comply with contraception and reproduction restrictions; able and willing to provide informed consent. | Seated SBP ≥180 or DBP ≥110 mm Hg; BMI >45 kg/m²; unstable medical/surgical conditions; severe kidney (eGFR <45 mL/min/1.73 m²) or heart disease (NYHA III/IV, recent MI/stroke/AF); uncontrolled diabetes (HbA1c >9.5%); pregnancy/breastfeeding; secondary hypertension (except eligible primary aldosteronism or sleep apnea); hypersensitivity to study drug; recent participation in other investigational studies. |
| **HALO, 2023** | Stable background antihypertensive therapy ≥8 weeks; mean seated SBP ≥140 mm Hg (≥130 if diabetic); able to adhere to study and background medications; sTable GLT2 inhibitor if applicable; agrees to contraception requirements. | Seated SBP ≥180 mm Hg; BMI >50 kg/m²; non-hypertension use of alpha/beta blockers; unable/unwilling to stop MRA or potassium-sparing diuretic; eGFR <30 mL/min/1.73 m²; NYHA III/IV heart failure; recent stroke, TIA, ACS, or heart failure hospitalization (<6 months); major cardiac surgery <6 months; chronic permanent AF; uncontrolled diabetes (HbA1c >10%); planned dialysis/kidney transplant; prior organ/cell transplant; serum sodium <130 mEq/L; serum potassium <3.5 or >5 mEq/L; abnormal WBC; positive HIV/HCV/HBV; high alcohol intake (≥14 drinks/week). |
| **LAUNCH-HTN, 2025** | Written informed consent; age ≥ 18 years; screening and randomization AOBP SBP ≥ 135 and ≤ 180 mm Hg with DBP ≥ 65 and ≤ 110 mm Hg, or DBP ≥ 90 and ≤ 110 mm Hg; treatment with 2–5 stable antihypertensive medications for ≥ 1 month before screening, including a thiazide or thiazide-like diuretic (stable ≥ 4 weeks before randomization); hypertension duration ≥ 6 months; morning serum cortisol 3–22 μg/dL at screening; BMI ≥ 18 kg/m²; arm circumference < 52 cm; agreement to effective contraception if of childbearing potential; willingness and ability to comply with study procedures and visits. | Pregnant or breastfeeding, hypersensitivity to lorundrostat, eGFR <45 mL/min/1.73 m², potassium >5.0 mmol/L or >4.8 mmol/L at randomization, sodium <135 mmol/L or history of hyponatremia, white-coat hypertension or autonomic dysfunction, night-shift work >14 days consecutively or >14 days/month, secondary hypertension (except sleep apnea or primary aldosteronism if MRA discontinued), recent HF, MI, stroke, or TIA (<6 months), uncontrolled diabetes (HbA1c >9%) or severe hypoglycemia history, major surgery <4 weeks prior or planned during study, recent malignancy (<5 years, except treated skin cancer or carcinoma in situ), recent MRA or ENaC inhibitor use, drug or alcohol abuse within 1 year, or other conditions per investigator judgment. |
| **FigHTN, 2025** | Adults with mean seated SBP ≥140 mm Hg, mild-to-severe CKD, elevated UACR, and on maximally tolerated ACEi or ARB therapy. | Type 1 diabetes; unwilling/unable to discontinue MRA or potassium-sparing diuretic; SBP >180 mm Hg or DBP >110 mm Hg during screening; BMI >50 kg/m²; bilateral renal artery stenosis ≥70%; recent or planned dialysis/kidney transplant; NYHA class III/IV heart failure or recent hospitalization for HF; recent stroke, TIA, ACS, or hypertensive encephalopathy; severe LV outflow obstruction; planned or recent major cardiac surgery; prior organ/cell transplant; hypersensitivity to CIN-107; recent immunotherapy for CKD; unstable medical/surgical conditions; abnormal electrolytes (serum sodium/potassium) or liver function; eGFR <25 or >75 mL/min/1.73 m²; uncontrolled diabetes (HbA1c >10.5%); HIV/HBV/HCV positive; heavy alcohol intake (>14 drinks/week). |
| **Target-HTN, 2023** | Adults ≥18 years and nonpregnant women, SBP ≥130 mm Hg, on ≥2 stable antihypertensives, adequate renal function (eGFR ≥60 mL/min/1.73m²), normal potassium and sodium, informed consent. | Use of MRA, ENaC inhibitors, ACEi+ARB combination, K⁺ <3.0 or >4.8–5.2 mEq/L, Na⁺ <135 mEq/L, eGFR <60 mL/min/1.73 m², uncontrolled diabetes (HbA1c ≥9%), recent major CV events (MI, stroke, unstable angina, AF), severe hypertension (SBP ≥175 or DBP ≥100), orthostatic hypotension, major comorbidities, interfering drugs (NSAIDs, strong CYP3A modulators, PDE5 inhibitors), chronic corticosteroid use, hypersensitivity to study drug, pregnancy. |
| **Tuttle, 2024** | Adults ≥18 years with CKD (±T2DM), eGFR 30–<90 mL/min/1.73 m², UACR 200–<5,000 mg/g, BMI 18.5–<50 kg/m², serum potassium ≤4.8 mmol/L, mean SBP 110–160 mm Hg and DBP 65–110 mm Hg, stable background antihypertensive therapy including ACEi or ARB (not both) for ≥4 weeks; women of childbearing potential and men with WOCBP partners must use effective contraception. | Pregnancy or nursing, use of MRAs/potassium-sparing diuretics or RAS-modifying drugs recently, type 1 diabetes, severe cardiac conduction abnormalities or uncompensated heart failure, kidney failure/dialysis/transplant, active or recent malignancy, significant liver disease, other medical conditions increasing risk or interfering with trial, high risk for ketoacidosis. |
| **BaxHTN, 2025** | Adults with mean seated SBP ≥ 140 and ≤170 during office visit, despite treatment with MTD of either two antihypertensive medications (for uncontrolled hypertension) or three or more medications (for resistant hypertension), including a diuretic, for at least 4 weeks before screening. | Mean seated SBP ≥ 170 mm Hg or DBP ≥110 mm Hg, current or prior treatment with an ARB and an ACEi, both taken simultaneously; Serum sodium < 135 mmol/L; known secondary hypertension; NYHA class IV; history of stroke, ACS, hypertensive encephalopathy, or hospitalization for heart failure in the last 6 months. |

***Abbreviations*:** ACEi: Angiotensin-Converting Enzyme Inhibitor; ACS: Acute Coronary Syndrome; ABPM: Ambulatory Blood Pressure Monitoring; AF: Atrial Fibrillation; AOBP: Automated Office Blood Pressure; ARB: Angiotensin II Receptor Blocker; BMI: Body Mass Index; CKD: Chronic Kidney Disease; CV: Cardiovascular; DBP: Diastolic Blood Pressure; DKA: Diabetic Ketoacidosis; eGFR: estimated Glomerular Filtration Rate; ENaC: Epithelial Sodium Channel; HbA1c: Hemoglobin A1c; HF: Heart Failure; HBV: Hepatitis B Virus; HCV: Hepatitis C Virus; HIV: Human Immunodeficiency Virus; LV: Left Ventricular; MI: Myocardial Infarction; MRA: Mineralocorticoid Receptor Antagonist; MTD: Maximally Tolerated Dose; NYHA: New York Heart Association; NSAID: Nonsteroidal Anti-inflammatory Drug; PDE5: Phosphodiesterase Type 5; SBP: Systolic Blood Pressure; SGLT2: Sodium-Glucose Cotransporter-2; TIA: Transient Ischemic Attack; UACR: Urinary Albumin-to-Creatinine Ratio; WBC: White Blood Cell; WOCBP: Women of Childbearing Potential.

# Supplemental Table 4. Characteristics of included studies

| **Study** | **Baseline antihypertensive drugs** | **Adherence** | **Run-in Requirements** |
| --- | --- | --- | --- |
| **ADVANCE-HTN,**  **2025** | Placebo group:  3-drugs: 34 (36%)  2-drugs: 61 (64%)  Lorundrostat all doses:  3-drugs: 72 (38%)  2-drugs: 118 (62%) | Not reported. | Participants stopped prior antihypertensives and received olmesartan 40 mg ± indapamide 2.5 mg or HCTZ 25 mg; for those on 3–5 drugs, amlodipine 10 mg was added, with lower doses (olmesartan 20 mg, amlodipine 5 mg) allowed per investigator. All received placebo during a 3-week single-blind run-in. ABPM performed after 3 weeks; participants with SBP 130–180 mm Hg or DBP >80 mm Hg were randomized to placebo or lorundrostat (50 mg stable or 50→100 mg titration). |
| **BrigHTN, 2023** | At baseline, most patients were taking 3 antihypertensive agents (58–70%), followed by 4 agents (15-28%), 5 agents (7-12%), and 2, 6-8 agents in smaller proportions. All patients (100%) were taking a diuretic. Beta-blockers were used by 61%, calcium-channel blockers by 68%, and ACE inhibitors or ARBs by 93% of patients. | Adherence was assessed via pill counts during the run-in period, with dosing informed by prior aldosterone-lowering studies. | After up to 8 weeks of screening, eligible patients entered a 2-week single-blind run-in to assess adherence. Patients with ≥70% adherence to each antihypertensive and placebo, and seated BP ≥130/80 mm Hg, were randomized. |
| **HALO, 2023** | Not reported. | Adherence was assessed during a 4-week run-in period; method not reported. | Up to 4-week run-in period conducted to confirm adherence to background antihypertensive medications and placebo. Of 631 screened patients, 382 (60.5%) failed screening. 249 patients were randomized to 4 treatment groups. |
| **LAUNCH-HTN,**  **2025** | Lorundrostat all doses:  2 agents: 319 (39.3%), ≥3 agents: 492 (60.6%)  Thiazide or thiazide-like diuretic: 779 (96.1%)  ACE inhibitor or ARB: 704 (86.8%)  Calcium channel blocker: 418 (51.4%)  Placebo:  2 agents: 113 (41.5%), ≥3 agents: 159 (58.5%)  Thiazide or thiazide-like diuretic: 259 (95.2%)  ACE inhibitor or ARB: 225 (82.7%)  Calcium channel blocker: 135 (49.6%) | Adherence was assessed during a 2-week run-in period; method not reported. | The study included a 2-week single-blind placebo run-in before randomization to assess adherence and ensure participants continued taking their prescribed antihypertensive medications. |
| **FigHTN, 2025** | Baxdrostat all doses:  ACE inhibitor: 48 (37%)  ARB: 78 (60%)  Diuretic: 54 (42%)  Placebo:  ACE inhibitor: 21 (32%)  ARB: 40 (61%)  Diuretic: 31 (47%) | Adherence was assessed calculating pill counts. | After screening (albuminuria confirmation, renal function, BP, and safety labs), eligible patients entered a mandatory 2-week single-blind placebo run-in while continuing stable background antihypertensive therapy. Eligibility was reassessed before randomization. |
| **Target-HTN, 2023** | At baseline, about half of the patients in the intervention group were taking two antihypertensive agents (51.9%), while 48.1% were on three or more, compared with 56.7% and 43.3% in the placebo group. Thiazide-type diuretics were used by 57.1% of patients in the intervention group and 53.3% in the placebo group, whereas ACE inhibitors or ARBs were used by 78.9% and 73.3%, respectively. | Adherence was assessed during a run-in period; method not reported. | Participants underwent a 2-4-week pre-screening period followed by a 2-week single-blind placebo run-in while continuing stable background antihypertensive therapy, with BP monitoring by AOBP and ABPM to confirm eligibility, assess adherence, and standardize measurements, after which they were randomized to placebo or lorundrostat at different doses, with weekly assessments for 8 weeks and a final follow-up visit 2–4 weeks later. |
| **Tuttle, 2024** | At baseline, two thirds of patients in the placebo group (67%) and approximately 70% of those in the intervention group were receiving an ARB. ACE inhibitors were used by 34% of patients in the placebo group and by about 30% in the intervention group. | Adherence was assessed during a run-in period; method not reported. | Participants underwent an 8-week run-in period before randomization. Aldosterone was measured during run-in, and cortisol at screening. ACTH challenge testing was performed at screening and during the study if cortisol levels were low or if participants showed signs of adrenal insufficiency. |
| **BaxHTN, 2025** | At baseline, patients were receiving 2–5 background antihypertensive medications. In the placebo group, 27% were on 2 drugs, 42% on 3 drugs, 27% on 4 drugs, and 5% on ≥5 drugs. In the intervention group, 27% were on 2 drugs, 40% on 3 drugs, 24% on 4 drugs, and 9% on ≥5 drugs. Most patients in both groups were receiving an ACE inhibitor or ARB (89% placebo, ≈90% intervention), beta- blockers (34% placebo, 31–37% intervention), calcium channel blockers (67% placebo, 70–72% intervention), and nearly all patients received a diuretic (100% placebo, 99–100% intervention) | Adherence was assessed by direct observation and pill count. | Eligible patients underwent a 2-week single-blind placebo run-in while continuing their stable medications; only those with seated SBP ≥135 mm Hg and ≥80% adherence to background therapy proceeded to randomization. |

**Abbreviations:** ABPM: Ambulatory Blood Pressure Monitoring; ACEi: Angiotensin-Converting Enzyme Inhibitor; ARB: Angiotensin II Receptor Blocker; AOBP: Automated Office Blood Pressure; ACTH: Adrenocorticotropic Hormone; BP: Blood Pressure; CKD: Chronic Kidney Disease; DBP: Diastolic Blood Pressure; HCTZ: Hydrochlorothiazide; SBP: Systolic Blood Pressure.

# Supplemental Table 5. Dose-response analyses

**Table 5A.** Dose-response relationship for systolic blood pressure

| **Dose (mg)** | **Mean (mmHg)** | **Lower 95% CrI** | **Upper 95% CrI** | **Probability MD > - 5 mmHg** |
| --- | --- | --- | --- | --- |
| 0.5 | -1.95 | -3.40 | -0.44 | 0% |
| 1.0 | -4.59 | -7.48 | -1.49 | 40.5% |
| 1.5 | -8.38 | -12.97 | -3.33 | 91% |
| 2.0 | -12.85 | -19.55 | -5.37 | 98% |

Probability threshold refers to a reduction above 5 mmHg in systolic blood pressure.

Abbreviations: **CrI:** credible interval; **MD:** mean difference for reduction in systolic blood pressure.

**Table 5B.** Dose-response relationship for systolic blood pressure

| **Dose (mg)** | **Mean (RR)** | **Lower 95% CrI** | **Upper 95% CrI** | **Probability RR >1.25** |
| --- | --- | --- | --- | --- |
| 0.5 | 1.856 | 0.888 | 3.944 | 0.810 |
| 1.0 | 2.963 | 0.806 | 11.306 | 0.857 |
| 1.5 | 3.683 | 0.582 | 23.539 | 0.952 |
| 2.0 | 3.940 | 0.279 | 51.397 | 0.905 |

Probability threshold refers to an increase of more than 25% in relative risk.

Abbreviations: **CrI:** credible interval; **RR:** relative risk.

**Table 5C.** Dose-response relationship for serious adverse events

| **Dose (mg)** | **Mean (RR)** | **Lower 95% CrI** | **Upper 95% CrI** | **Probability RR >1.25** |
| --- | --- | --- | --- | --- |
| 0.5 | 0.783 | 0.387 | 1.534 | 8.8% |
| 1.0 | 0.758 | 0.229 | 2.408 | 18.7% |
| 1.5 | 1.048 | 0.213 | 4.708 | 39.7% |
| 2.0 | 1.793 | 0.182 | 14.001 | 67.7% |

Probability threshold refers to an increase of more than 25% in relative risk.

Abbreviations: **CrI:** credible interval; **RR:** relative risk.

# Supplemental Table 6. Risk of bias assessment of RCTs with RoB-2 tool

| Study | D1 | D2 | D3 | D4 | D5 | Overall risk of bias |
| --- | --- | --- | --- | --- | --- | --- |
| **ADVANCE-HTN**  2025 | Low | Low | Low | Low | Low | Low |
| **BrigHTN**  2023 | Low | Low | Low | Low | Low | Low |
| **Launch-HTN**  2025 | Low | Low | Low | Low | Low | Low |
| **HALO**  2023 | Low | Low | Low | Low | Some concerns | Some   concerns |
| **Target-HTN**  2023 | Low | Low | Low | Low | Low | Low |
| **Tuttle**  2024 | Low | Low | Low | Low | Low | Low |
| **FigHTN**  2025 | Low | Low | Some concerns | Low | Some concerns | Some  concerns |
| **BaxHTN** 2025 | Low | Low | Low | Low | Low | Low |

Domains:

D1: Bias from randomization process.

D2: Bias due to deviations from intended interventions.

D3: Bias due to missing outcome data.

D4: Bias in measurement of the outcomes.

D5: Bias in selection of the reported results

# Supplemental Figure 1. Efficacy outcomes

**Figure 1A.** Systolic blood pressure target


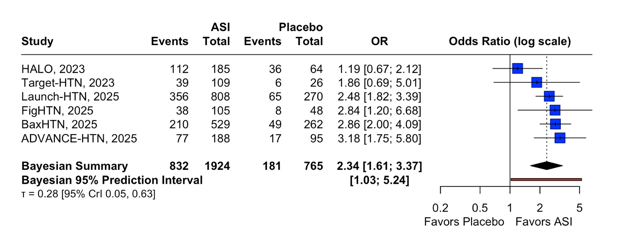


**Figure 1B.** Systolic blood pressure for low doses of ASIs

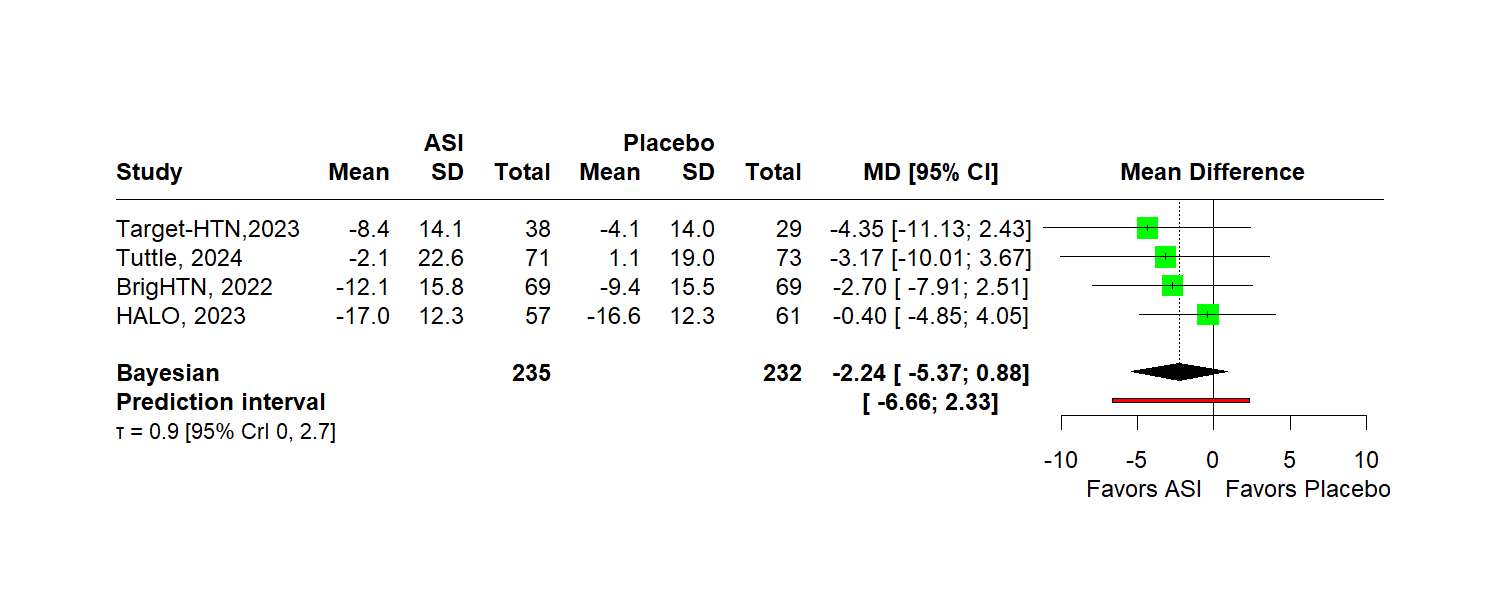


**Figure 1C.** Systolic blood pressure for intermediate doses of ASIs

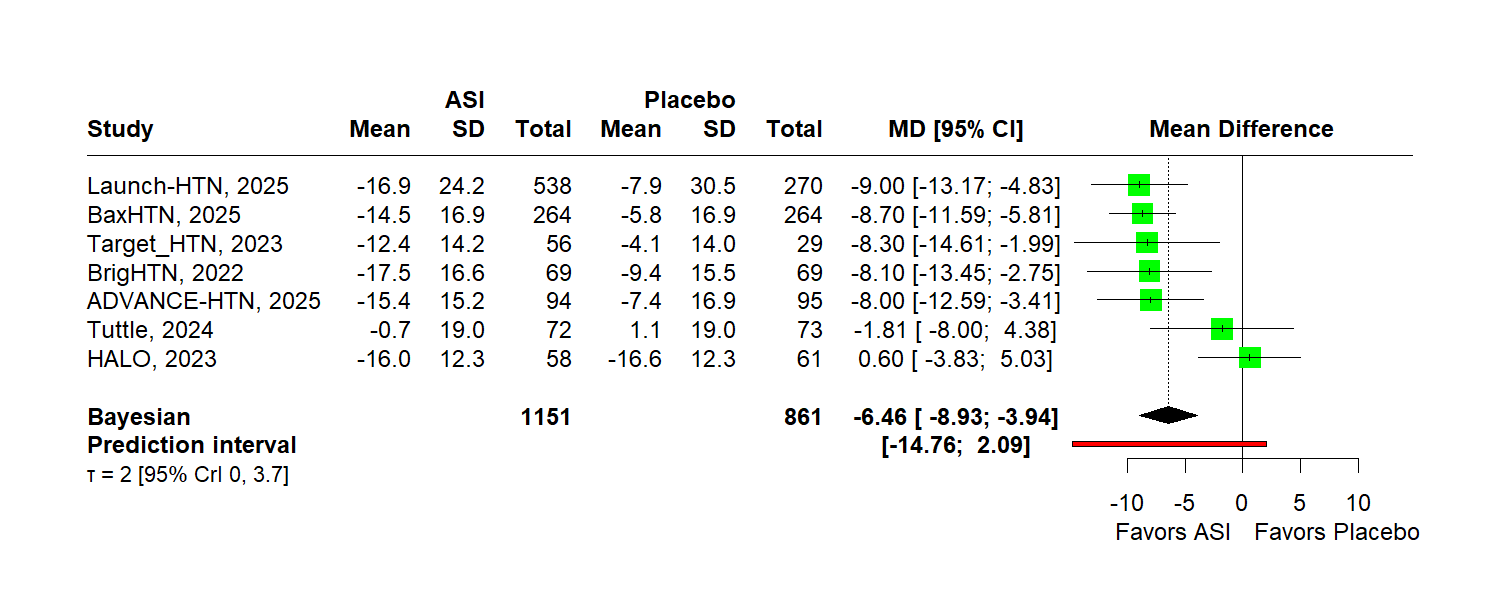


**Figure 1D.** Systolic blood pressure for high doses of ASIs

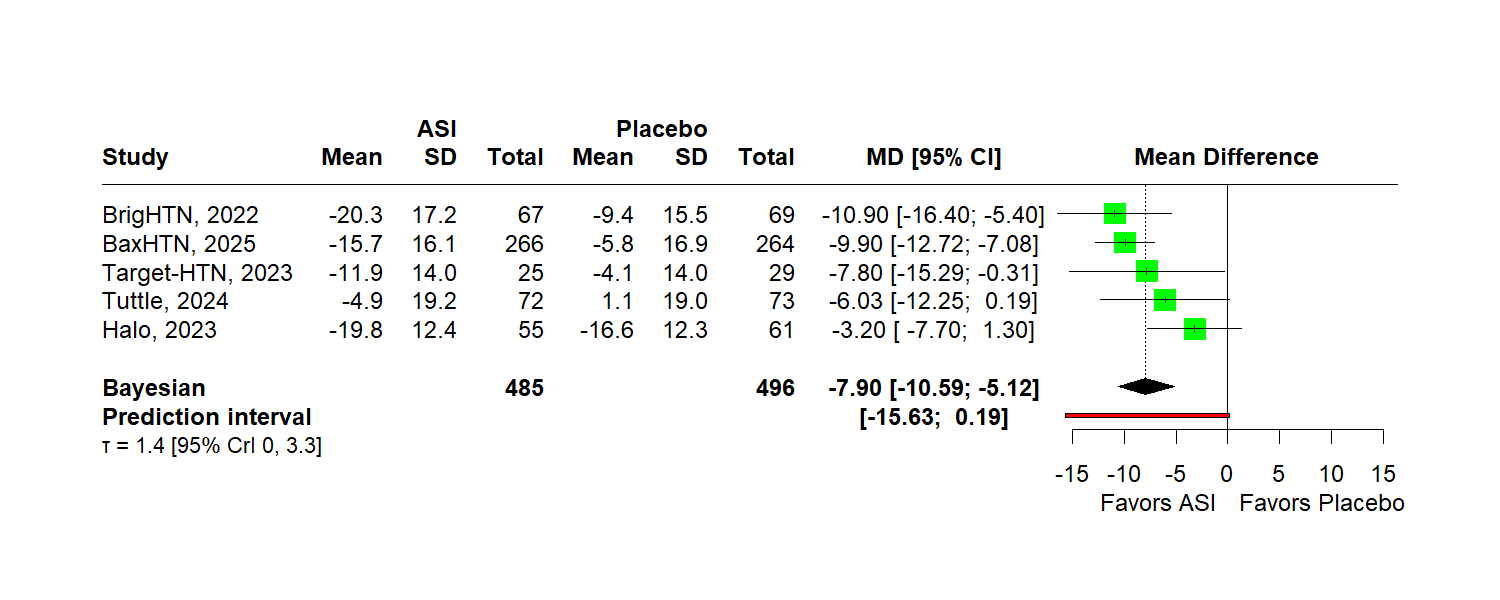


# Supplemental Figure 2. Safety outcomes

Odds ratios (ORs) were obtained by exponentiating the posterior log ORs using full model precision. Minor differences may occur if exponentiation is applied to rounded values displayed in the figure.

**Figure 2A.** Hypertensive urgency

**
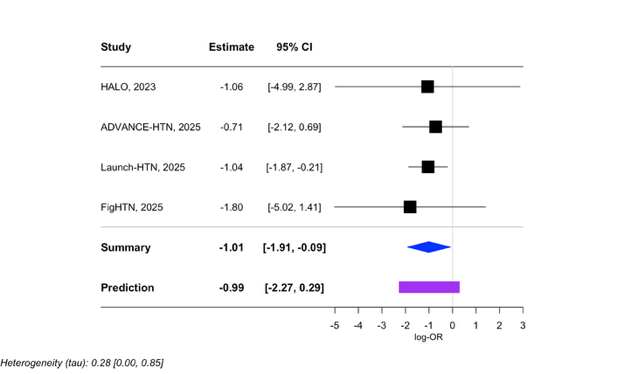
**

The summary log-odds ratio (OR) of -1.01 corresponds to an OR of 0.36 (95% CrI, 0.13-0.91).

*Abbreviation:* CrI, credible interval.

**Figure 2B.** Hyperkalemia


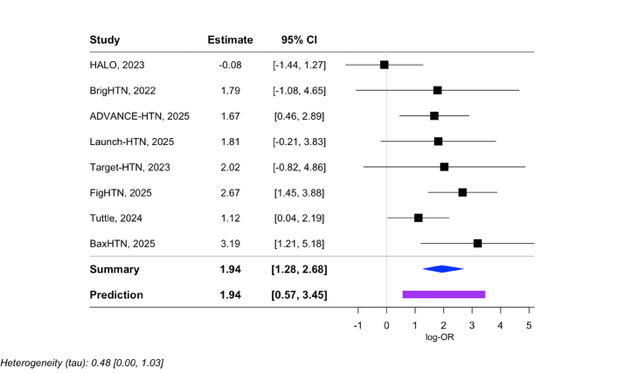


The summary log-odds ratio (OR) of 1.94 corresponds to an OR of 7.1 (95% CrI, 3.56–15.2).

*Abbreviation:* CrI, credible interval.

**Figure2C.** Severe hyperkalemia (K ≥ 6.0 mmol/L)


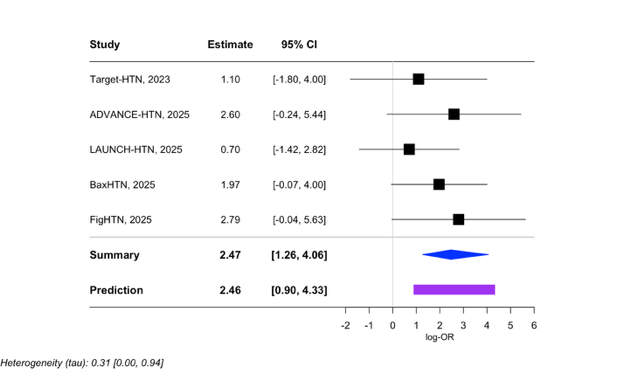


The summary log-odds ratio (OR) of 2.47 corresponds to an OR of 12.55 (95% CrI, 3.52–61.9). **Abbreviation:** CrI, credible interval.

**Figure 2D.** Hyponatremia


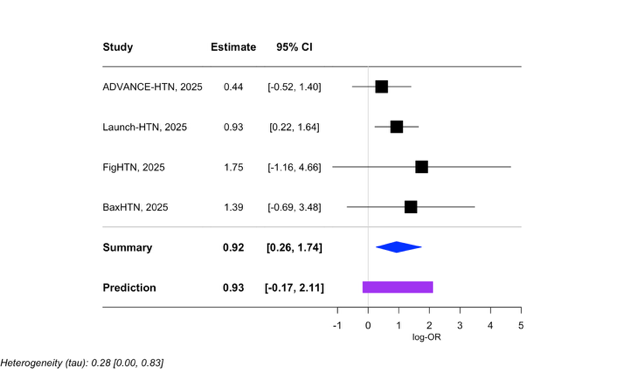


The summary log-odds ratio (OR) of 0.92 corresponds to an OR of 2.6 (95% CrI, 1.25–5.98. *Abbreviation:* CrI, credible interval.

**Figure 2E.** Hypotension


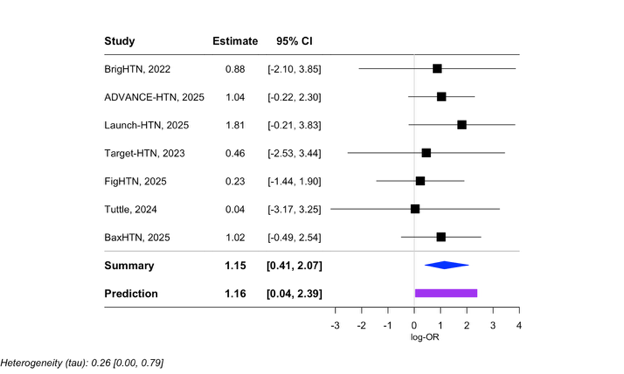


The summary log-odds ratio (OR) of 1.15 corresponds to an OR of 3.28 (95% CrI, 1.43–8.16. *Abbreviation:* CrI, credible interval.

**Figure 2F.** Serious adverse events

**
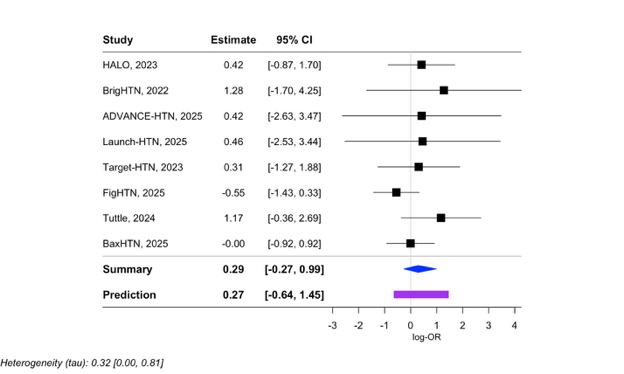
**

The summary log-odds ratio (OR) of 0.05 corresponds to an OR of 1.4 (95% CrI, 0.77–2.85). Abbreviation: CrI, credible interval.

**Figure 2G.** Drug-related serious adverse events


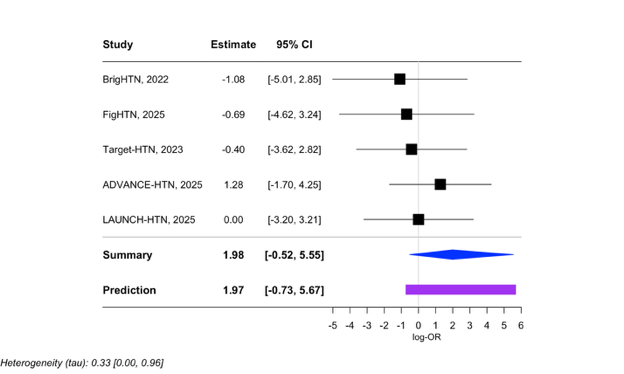


The summary log-odds ratio (OR) of 1.98 corresponds to an OR of 8.33 (95% CrI, 0.67–232.75). *Abbreviation:* CrI, credible interval.

**Figure 2H.** All-cause mortality


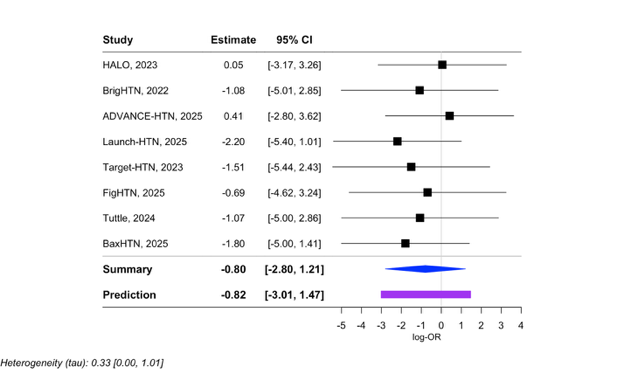


The summary log-odds ratio (OR) of –0.80 corresponds to an OR of 0.45 (95% CrI, 0.06–3.23. *Abbreviation:* CrI, credible interval.

**Figure 2I.** Adrenal insufficiency


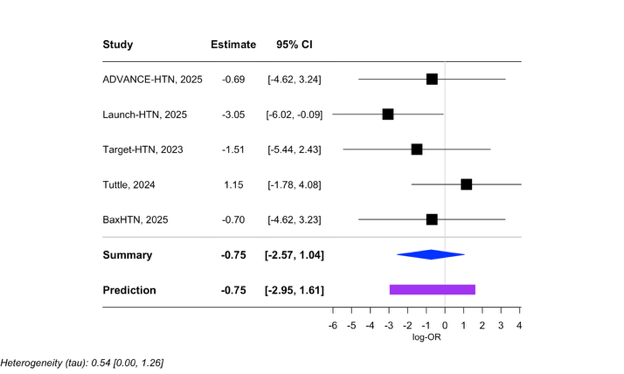

The summary log-odds ratio (OR) of –0.75 corresponds to an OR of 0.47 (95% CrI, 0.08–3.1. *Abbreviation:* CrI, credible interval.

**Figure 2J.** Any arrhythmia


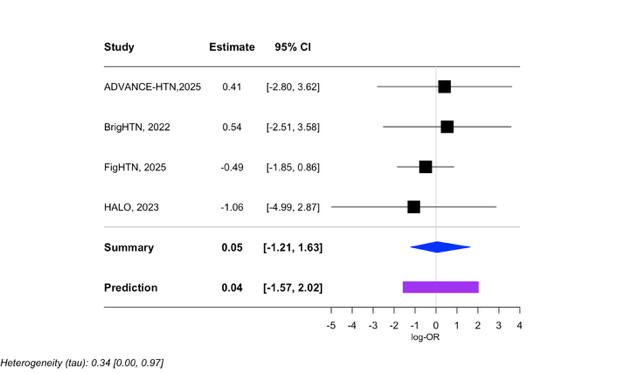


The summary log-odds ratio (OR) of 0.05 corresponds to an OR of 1.07 (95% CrI, 0.30–4.8). *Abbreviation:* CrI, credible interval.

**Supplemental Figure 3: Subgroup analysis by drug type (Baxdrostat vs. Lorundrostat)**

**Figure 3A.** Diastolic blood pressure


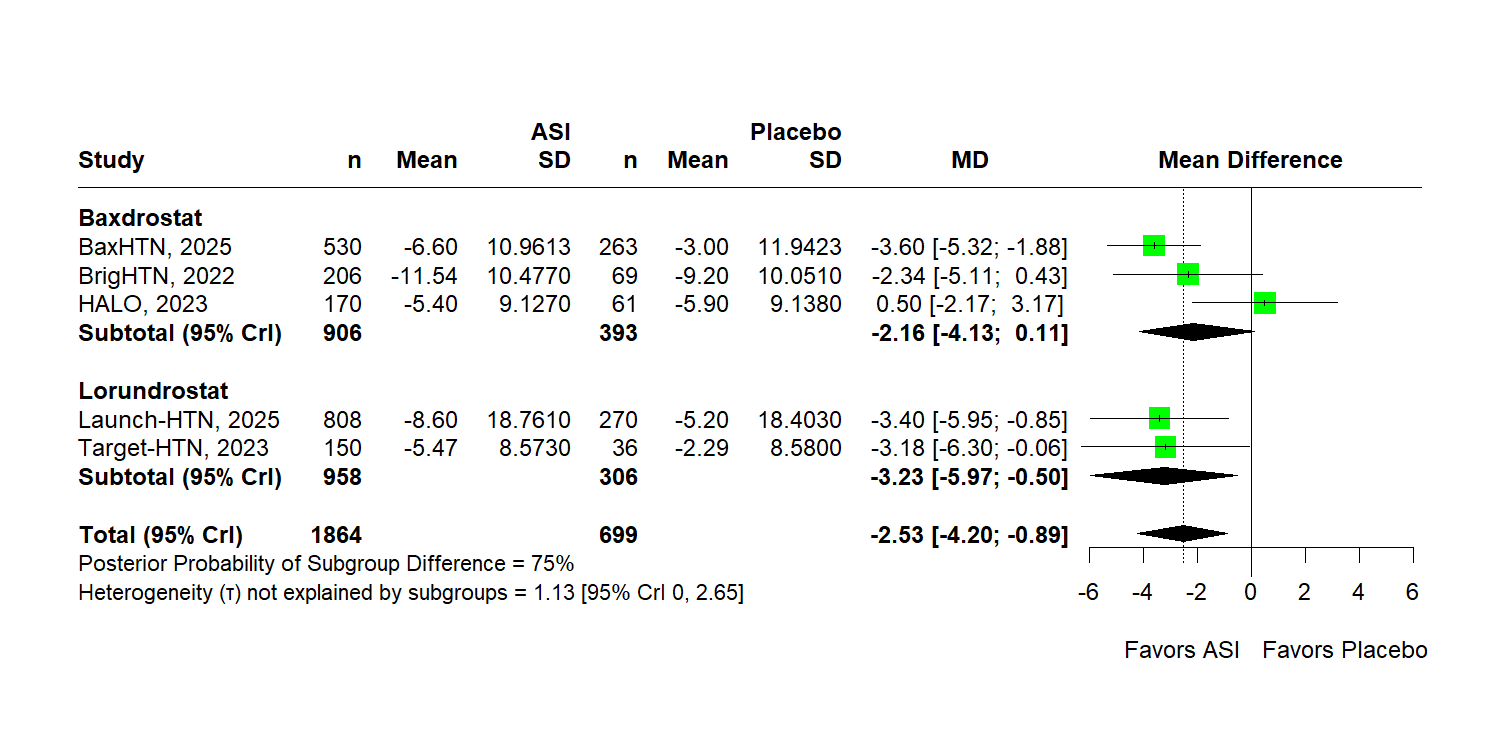


**Figure 3B.** Systolic blood pressure target


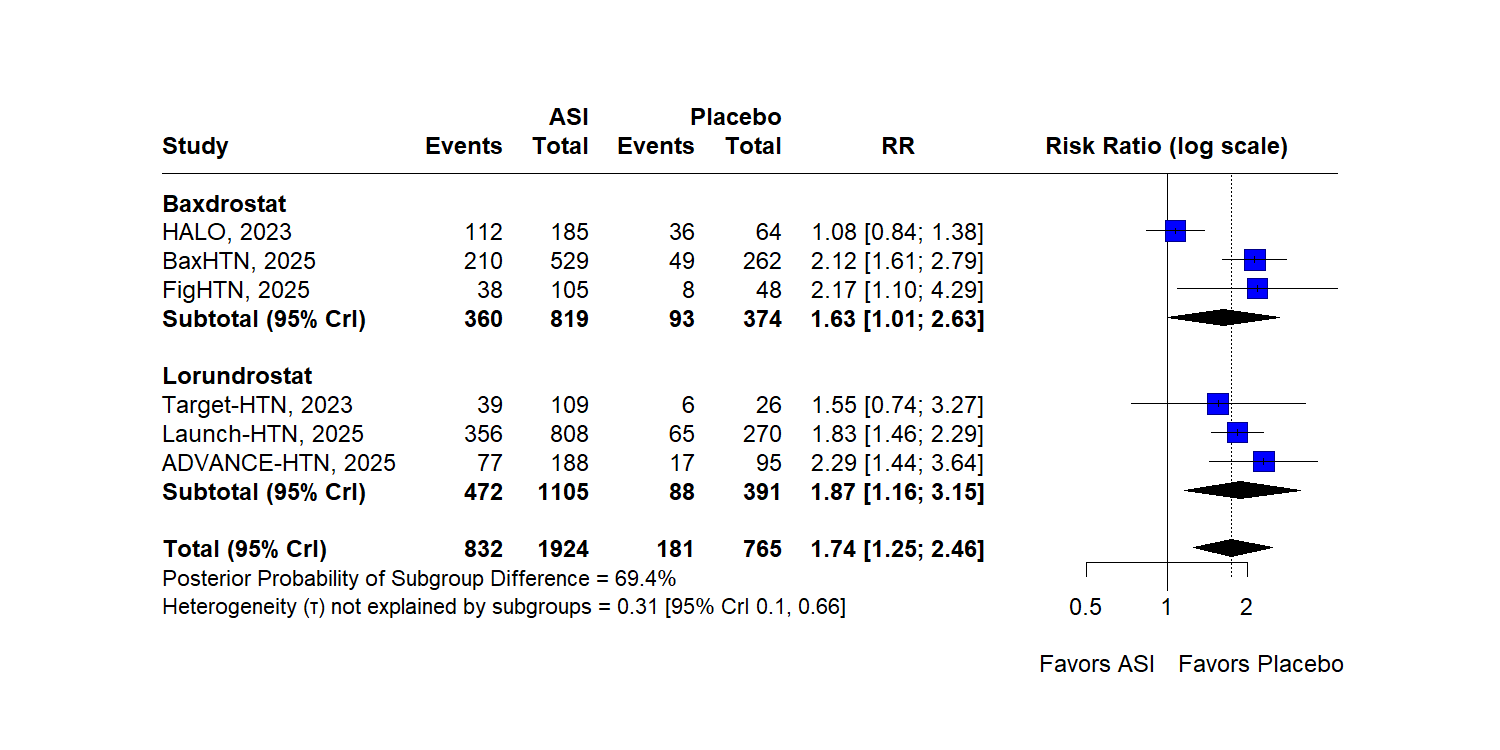


# Supplemental Figure 4: Leave-one-out analysis

**Figure 4A.** Systolic blood pressure


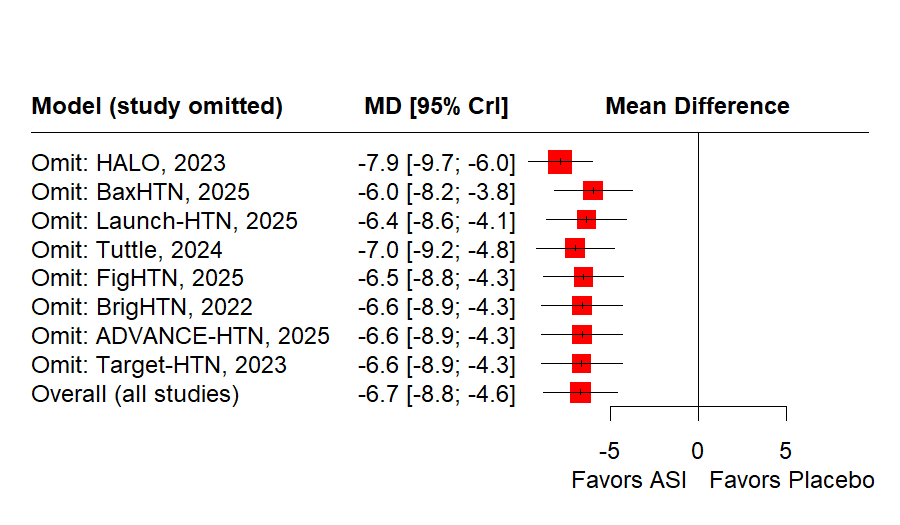


**Figure 4B.** Diastolic blood pressure


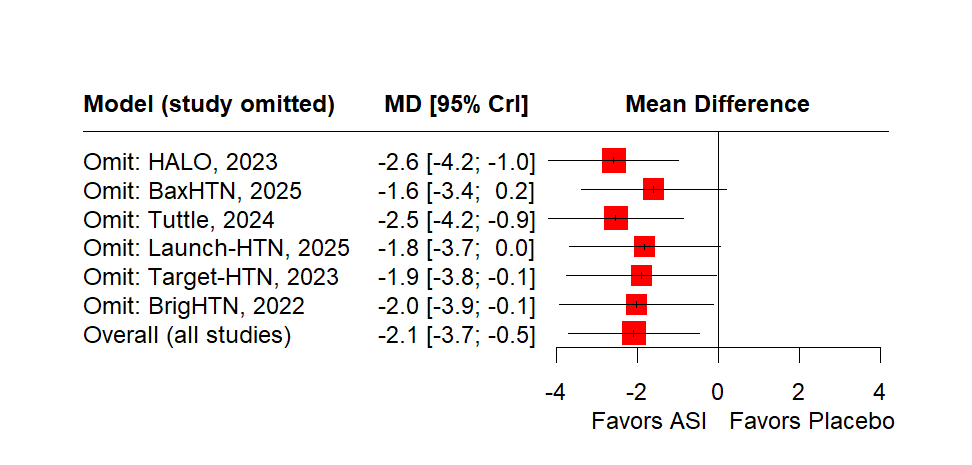


**Figure 4C.** Systolic blood pressure target


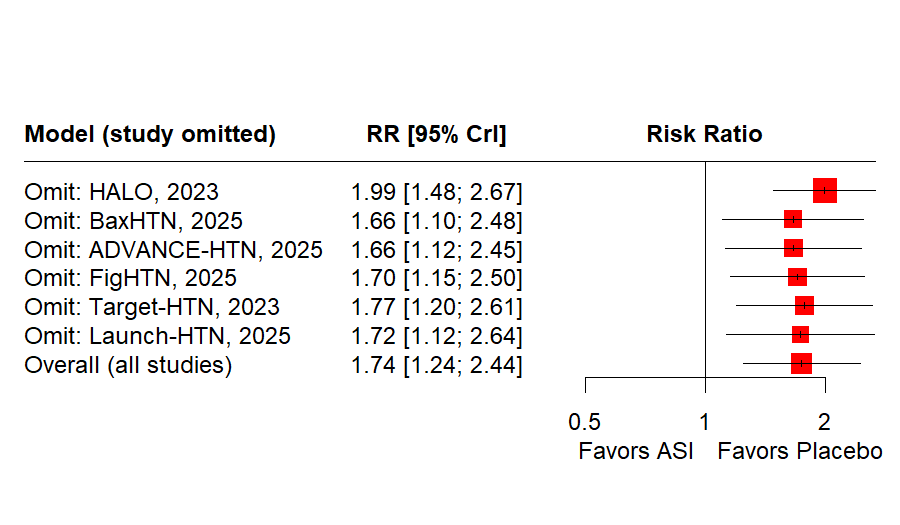


# Supplemental Figure 5. Sensitivity analysis for systolic blood pressure

# Figure 5A: Overall Effect


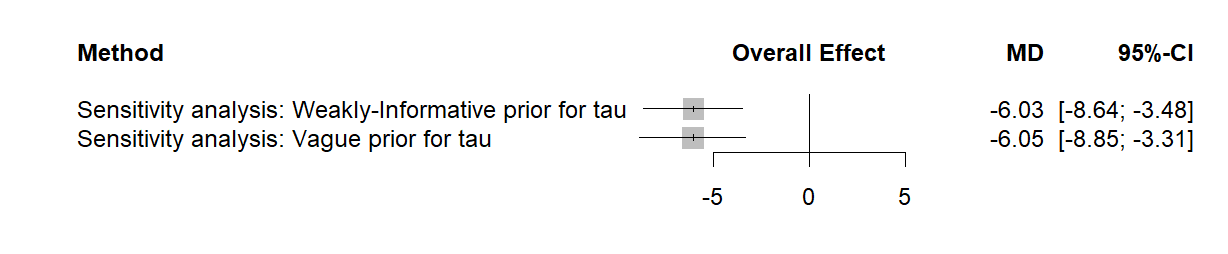


# Figure 5B. Posterior predictive distribution

**
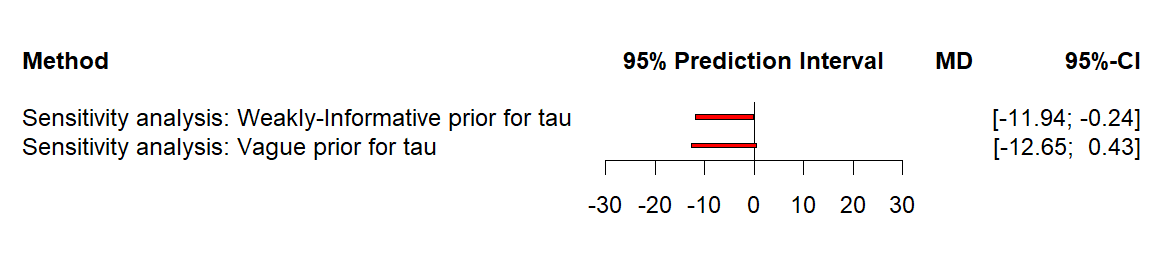
****Figure 5C.** Posterior predictive distribution sensitivity analysis of overall effect (green) and between-study heterogeneity (blue)

**
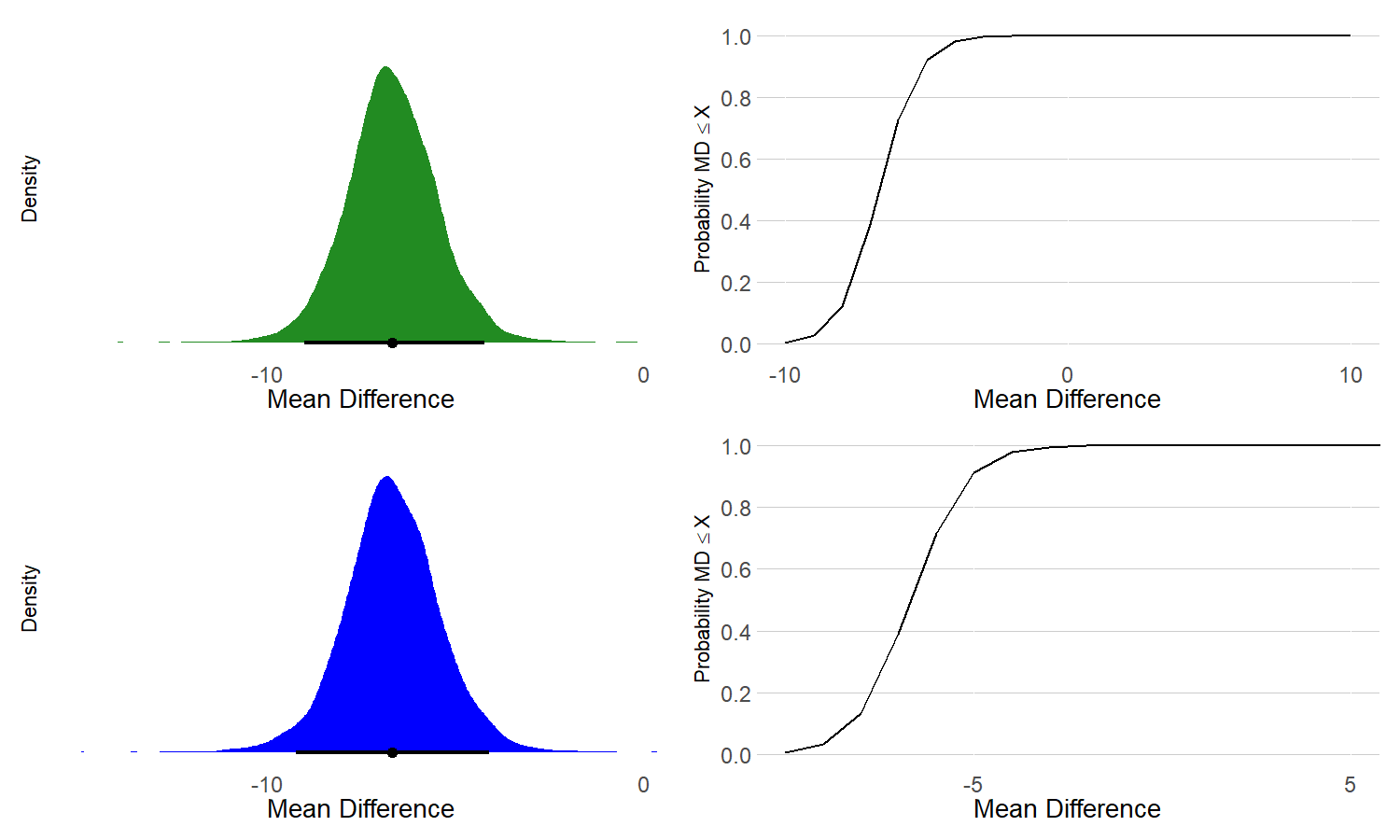
**

**Figure 5D.** SBP sensitivity analysis using a non-informative prior for overall effect centered at zero with reduced variance


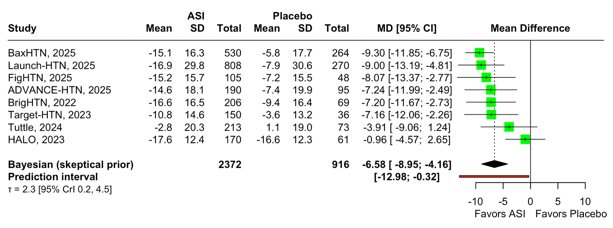


For this sensitivity analysis, mu was modeled using a skeptical weakly-informative prior, specified as Normal (0, 5.1²), reflecting ±20 mmHg as the approximate 95 percent prior interval. The prior for tau remained weakly-informative, specified as Half-Normal (1.67), consistent with the main analysis.

# Supplemental Figure 6. Dose-response analyses

**Figure 6A.** Systolic blood pressure

**
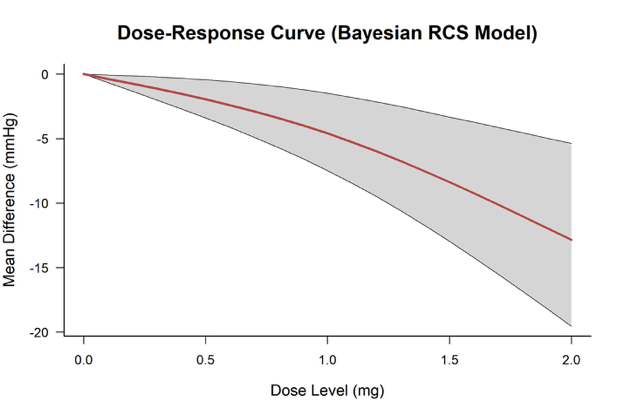
**

**Figure 6B.** Hyperkalemia

**
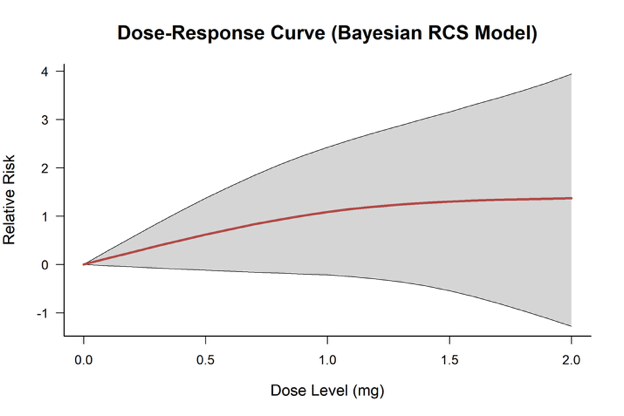
**

**Figure 6C.** Serious adverse events

**
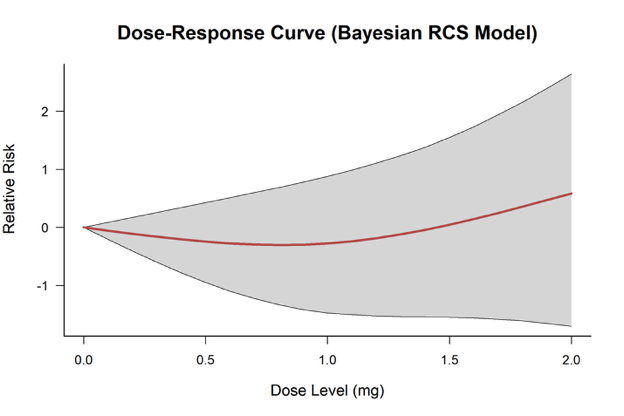
**

# Supplemental Figure 7. Funnel plot for systolic blood pressure


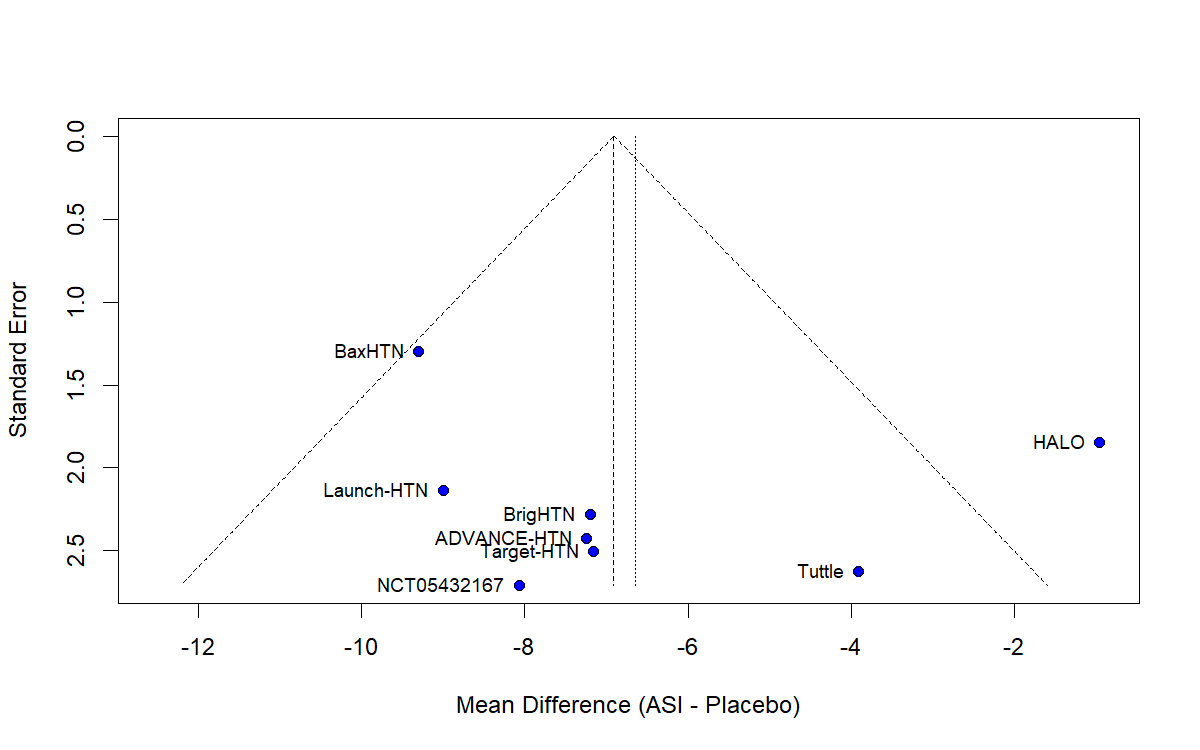


# Supplemental Figure 8. Egger’s test for systolic blood pressure


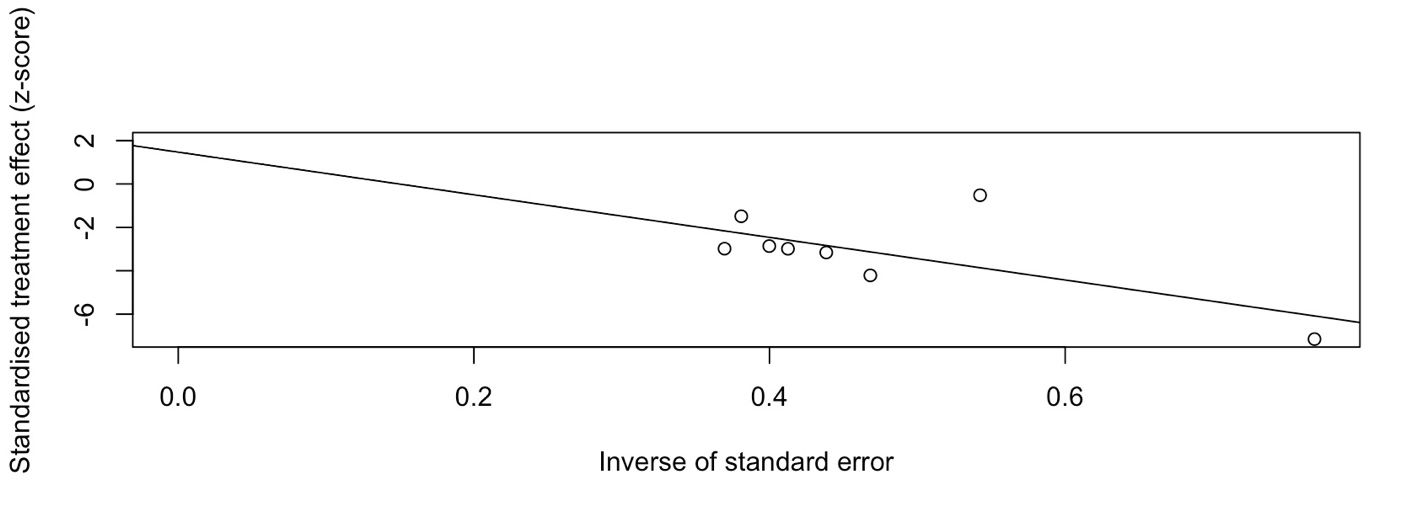


Test result: t = 0.66, df = 6, p-value = 0.532

Bias estimate: 1.47 (SE = 2.22)

*Abbreviations:* t, t-statistic from the regression test; df, degrees of freedom; SE, standard error.

**References**

1. Turner RM, Jackson D, Wei Y, Thompson SG, Higgins JPT. Predictive distributions for between‐study heterogeneity and simple methods for their application in Bayesian meta‐analysis. *Statistics in Medicine*. 2015;34(6):984-998. doi:10.1002/sim.6381

2. Röver C. Bayesian Random-Effects Meta-Analysis Using the **bayesmeta** *R* Package. *J Stat Soft*. 2020;93(6). doi:10.18637/jss.v093.i06

3. Röver C, Bender R, Dias S, et al. On weakly informative prior distributions for the heterogeneity parameter in Bayesian random‐effects meta‐analysis. *Research Synthesis Methods*. 2021;12(4):448-474. doi:10.1002/jrsm.1475

4. Spiegelhalter DJ, Abrams KR, Myles JP. *Bayesian Approaches to Clinical Trials and Health‐Care Evaluation*. 1st ed. Wiley; 2003. doi:10.1002/0470092602

5. Günhan BK, Röver C, Friede T. Random‐effects meta‐analysis of few studies involving rare events. *Research Synthesis Methods*. 2020;11(1):74-90. doi:10.1002/jrsm.1370
